# Advances in micro and nanoengineered surfaces for enhancing boiling and condensation heat transfer: a review

**DOI:** 10.1039/d2na00669c

**Published:** 2022-12-22

**Authors:** Nithin Vinod Upot, Kazi Fazle Rabbi, Siavash Khodakarami, Jin Yao Ho, Johannes Kohler Mendizabal, Nenad Miljkovic

**Affiliations:** a Department of Mechanical Science and Engineering, University of Illinois at Urbana-Champaign Urbana IL 61801 USA nmiljkov@illinois.edu; b School of Mechanical and Aerospace Engineering, Nanyang Technological University 50 Nanyang Avenue Singapore 639798 Republic of Singapore; c Department of Electrical and Computer Engineering, University of Illinois at Urbana-Champaign Urbana IL 61801 USA; d Materials Research Laboratory, University of Illinois at Urbana-Champaign Urbana IL 61801 USA; e International Institute for Carbon Neutral Energy Research (WPI-I2CNER), Kyushu University 744 Moto-oka, Nishi-ku Fukuoka 819-0395 Japan

## Abstract

Liquid–vapor phase change phenomena such as boiling and condensation are processes widely implemented in industrial systems such as power plants, refrigeration and air conditioning systems, desalination plants, water processing installations and thermal management devices due to their enhanced heat transfer capability when compared to single-phase processes. The last decade has seen significant advances in the development and application of micro and nanostructured surfaces to enhance phase change heat transfer. Phase change heat transfer enhancement mechanisms on micro and nanostructures are significantly different from those on conventional surfaces. In this review, we provide a comprehensive summary of the effects of micro and nanostructure morphology and surface chemistry on phase change phenomena. Our review elucidates how various rational designs of micro and nanostructures can be utilized to increase heat flux and heat transfer coefficient in the case of both boiling and condensation at different environmental conditions by manipulating surface wetting and nucleation rate. We also discuss phase change heat transfer performance of liquids having higher surface tension such as water and lower surface tension liquids such as dielectric fluids, hydrocarbons and refrigerants. We discuss the effects of micro/nanostructures on boiling and condensation in both external quiescent and internal flow conditions. The review also outlines limitations of micro/nanostructures and discusses the rational development of structures to mitigate these limitations. We end the review by summarizing recent machine learning approaches for predicting heat transfer performance of micro and nanostructured surfaces in boiling and condensation applications.

## Abstract 2

This review highlights recent developments in improving thermal-hydraulic performance through two phase heat transfer facilitated by microstructured and nanostructured surfaces.

## Graphical abstract

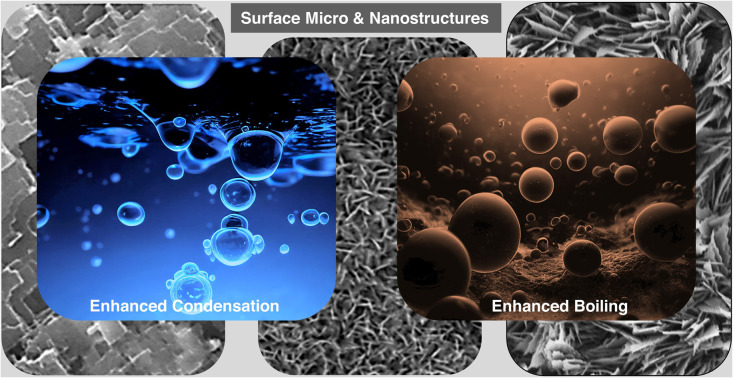

## 1. Introduction

Nanoengineered surfaces are at the forefront of next generation energy-efficient thermal management systems due to their immense potential to enhance phase change heat transfer. In particular, the presence of surface microstructures (structure characteristic length scale >1 μm) and nanostructures (structure characteristic length scale <1 μm) have the potential to alter surface chemistry and significantly influence solid–liquid–vapor interfacial dynamics to radically promote phase transition. Amongst the various phase change modes, liquid–vapor phase transitions, *i.e.*, boiling and condensation, are the most widely implemented in industrial systems such as in steam-based power plants,^1^ refrigeration cycles,^2^ seawater desalination installations,^3^ thermal management devices for electronics,^4–10^ and electrified transportation.^11–13^

Condensation occurs when vapor is converted to liquid by latent heat removal as it meets a subcooled surface. The introduction of functionalized micro/nanostructures enables the realization of discrete droplet formation during condensation (also known as dropwise condensation^14^) which is characterized by high heat removal rates as opposed to the conventional filmwise condensation mode which faces a fundamental limitation of the high thermal barrier imposed by conduction through the condensate film.^15–17^ Recently, the discovery of novel jumping droplet condensation mechanisms^18–20^ on micro and nanostructures that fundamentally differ from conventional dropwise condensation on smooth promoter coatings^21–23^ have also spurred renewed research interest. Similarly, boiling, a process of liquid-to-vapor conversion latent heat supply and bubble nucleation, growth and departure on a heated surface, can also be enhanced by the addition of micro and nanostructures on the boiling surface. These structures not only provide cavities of appropriate length-scale to activate bubble nucleation which improves heat transfer coefficient (HTC), but they also demonstrate potential to enable surface self-rewetting which delays the occurrence of the undesirable critical heat flux (CHF), a hydrodynamically unstable state.

Owing to the promising demonstration of micro/nanostructures to enhance boiling and condensation and their potential for improvements in two-phase system efficiency, extensive studies in the field of phase change heat transfer have been conducted resulting in a large collection of experimental data. Many variations of micro- and nano-engineered surfaces (estimate to be as high as 1000) have also been introduced and characterized for a variety of substrates and materials. A review of the micro/nanostructure morphologies and their related thermal performance is, therefore, essential not only to identify better-performing structure morphologies and to ascertain the potential phase change mechanisms within these structures, but also to identify existing research gaps and important works that need to be conducted. Furthermore, considering the rapid advancements in nanofabrication in recent years, it is also timely to review and assess the recent micro and nanoscale visualization techniques implemented in phase change heat transfer applications and the new transport phenomena uncovered.

In this review, we discuss recent progress in micro/nanostructure enhanced boiling and condensation heat transfer by focusing on the influence of surface morphology on the transport mechanisms of heat and mass. Key thermal performance indicators such as boiling/condensation HTC, condensate flooding and boiling CHF of the various micro/nanostructured surfaces shall be collated and analyzed. We hope that through this review, we can identify rational approaches to modifying surface wettability through implementation of micro/nanostructures to enhance HTC and CHF under both quiescent and flow conditions. Existing reviews on boiling/condensation heat transfer focus on overall surface engineering such as micro/nanostructuring or wettability modification of smooth as well as structured surfaces for enhancing condensation and boiling.^24–27^ Unlike past reviews, this review not only covers the material development aspect of enhanced surfaces, but it also presents in depth discussion regarding the synergistic interaction of bubbles/droplets and structures and their influence on boiling/condensation mechanisms such as microscopic bubble/droplet growth dynamics and three-phase contact line evolution. When compared to previous reviews,^24–27^ both for boiling and condensation enhancement, we include sections focusing on non-flow (quiescent) conditions as well as flow conditions, where we discuss how different design of micro/nanostructured surfaces can be utilized for each specific condition. Apart from the commonly used fluids such as water, here the effectiveness of micro and nanostructured surfaces in promoting boiling and condensation of liquids having various surface tension *i.e.*, water, hydrocarbons and refrigerants commonly used in industry, are critically evaluated. Despite the development of a plethora of micro and nanostructures over the last decade, many surface fabrication methods are not scalable. Furthermore, the majority of surface micro/nanostructures are susceptible to degradation and surface contamination which prevents their implementation in long term commercial and industrial applications. Our review discusses potential applications and guidelines for scalable and durable implementation of micro/nanostructures at the industrial level. Furthermore, considering recent interest in machine learning application to predict phase change heat transfer performance, we include analysis and discussion of available machine learning approaches for both boiling and condensation applications.

## 2. Pool boiling

**Fig. 1:**
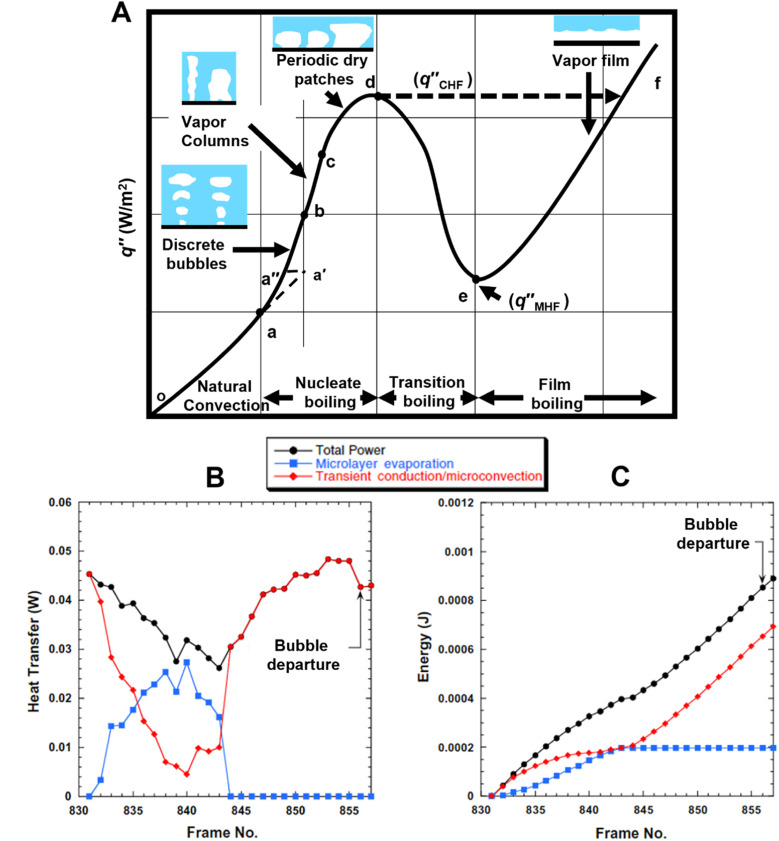
(A) Heat flux as a function of wall superheat for saturated pool boiling.^28^ Relative contributions of microlayer evaporation and transient conduction and microconvection to (B) local heat transfer rate and (C) total energy during nucleate pool boiling under a single bubble.^30^ Reproduced with permission from ref. 30. Copyright 2005 Elsevier.

The boiling curve for saturated pool boiling, a plot of heat flux against wall superheat, is shown in Fig. 1A.^28^ The region o-a corresponds to heat transfer by natural convection with no bubbles being formed. At point a, bubbles begin to form once the wall superheat has been raised to a sufficiently high level and this point is known as the onset of nucleate boiling (ONB). For highly wetting fluids which exhibit low surface tension, the ONB could be delayed to point a′ and is typically followed by numerous cavities being activated together which leads to a reduction in wall superheat at the same heat flux condition (region a′–a). Nucleate boiling begins from point a after ONB and isolated discrete bubbles control heat transfer in the initial region of nucleate boiling on increasing heat flux (a–b). Further increase in heat flux leads to activation of more nucleation sites and generation of more bubbles with bubble coalescence being observed. This region (b–c) is characterized by vapor columns with high heat transfer coefficients being exhibited in the fully developed nucleate boiling portion of the boiling curve. It is important to note that while the existence of vapor columns have been postulated in early pool boiling models, physical observation of such columns remain scarcely reported and is a topic that needs to be studied further.^29^ Continuous increase of heat flux will eventually lead to periodic dry patches being formed on the heater surface which are rewetted by the surrounding liquid (c–d) and this in turn leads to a reduction in the slope of the boiling curve which translates to reduction in heat transfer coefficients. Once the heater surface is covered with a vapor blanket, liquid is unable to rewet the surface and this point is known as the critical heat flux (CHF). The wall temperature drastically increases and can lead to heater burnout depending on the material. The region e–f corresponds to film boiling where the surface is covered with vapor without any liquid in direct contact and can be attained by reduction of heat flux. The region d–e in between nucleate boiling and film boiling regions is known as the transition region and is very unstable.

Since the nucleate boiling portion of the pool boiling curve results in the highest heat transfer coefficients, most applications involving pool boiling tend to focus on operation in this region. Within nucleate boiling, heat transfer primarily occurs through three modes: (1) microlayer evaporation, (2) transient conduction, and (3) microconvection. Microlayer evaporation refers to heat transfer due to evaporation of liquid underneath the growing vapor bubble and the interface of the vapor bubble. Transient conduction occurs as the liquid front advances to rewet the dry patch, while microconvection refers to heat transfer caused by disturbance of liquid layer adjacent to departing bubbles. Microlayer evaporation leads to eventual formation of dry spots on the heater surface and surrounding liquid rewets the surface leading to heat transfer due to transient conduction from the heater surface and microconvection. The relative contributions of the components of the bubble ebullition cycle have been investigated in the past,^30–32^ with many previous studies focused on microlayer evaporation contribution.^33–35^Fig. 1B and C displays the microlayer evaporation contribution and transient conduction/microconvection contribution during saturated pool boiling of FC-72 obtained with microheaters. The microlayer evaporation component was found to contribute up to 20% to the total heat transfer while the transient conduction and microconvective components dominate. While similar trends have been reported in other studies as well,^36,37^ well developed understanding of each of these three regions is essential for future pool boiling work.

### 2.1 Mechanisms governing micro/nanostructure enhanced heterogeneous pool boiling

In heterogeneous pool boiling, bubbles grow from pre-existing vapor embryos trapped in the cavities of a heated surface. The mechanisms driving bubble growth not only depends on the surface thermal conditions but also on the presence of suitable cavity size range for vapor entrapment and growth activation. By considering transient thermal boundary layer development during the bubble ebullition cycle, existing models have showed that bubble nucleation takes place only in submicron and micron scale cavities and the actual size range depends on the fluid properties such as surface tension and latent heat of vaporization.^38–40^ For instance, active bubble nucleation sites typically require the surface cavity radii to range between 0.01 and 1 μm for water,^41^ whereas for significantly lower surface tension and latent heat fluids such as dielectric fluids, cavity radii of 0.03 to 50 μm are found to be more desirable.^42^ While conventional unstructured “smooth” surfaces possess inherent cavities formed through the manufacturing process, the cavity sizes are undesirable for bubble nucleation resulting in high boiling incipience temperature and limited heat transfer.^43^ In this regard, surface micro/nanoengineering has the potential to the generate suitable surface cavities to increase nucleation size density for pool boiling enhancements and have received increasing attention in the last few decades.^44–47^ Furthermore, the wall superheat required for onset of nucleate boiling (ONB) has been shown to decrease with engineered nucleation sites,^48^ thus expanding the region of nucleate pool boiling and increasing HTC.

In addition to increasing the number of active nucleation sites, cavity geometry parameters such as width and depth also play an important role in improving heat transfer. A decrease in ONB superheat with increasing cavity depth and increased nucleation leading to HTC enhancements of 150% has been reported.^49^ However, an increase in cavity depth can also lead to smaller enhancements due to increased resistance to liquid replenishment, which in turn leads to heat transfer degradation,^50^ thus implying a non-monotonous relationship of cavity depth with heat transfer enhancements. Cavity sizing and cavity spacing are two other important characteristics to be considered when designing micro/nanoengineered surfaces. For instance, while increasing nucleation site density increases HTC due to higher bubble generation, this also leads to the overcrowding of bubbles on the heated surfaces which coalescences to form large vapor mushrooms and early occurrence of CHF.^51^ It has been shown that an increase in microcavity diameter along with a decrease in spacings between microcavities can lead to the undesirable effect of bubble overcrowding and reduce CHF.^52^ To overcome this issue, surfaces can be designed to enable differentiated bubble departure and liquid replenishment pathways, minimizing the resistance to bubble removal and facilitating rewetting of the heated surface.^53^ Also known as separation of liquid–vapor pathways, this approach is commonly adopted to enable HTC and CHF co-enhancement.^54–56^ Instead of having surfaces uniformly coated with micro/nanostructures, hybrid surfaces consisting of structured and plain regions are an effective strategy to facilitate separated liquid–vapor pathways.^57,58^ Biphilic surfaces can have increased heat transfer coefficients when compared to surfaces with uniform wettability due to efficient liquid and vapor transport.^59^ Furthermore, the creation of cavities of appropriate size can tune the bubble departure direction, *i.e.*, whether bubbles escape from top or side, relative to bubble departure diameter.^53^

**Fig. 2:**
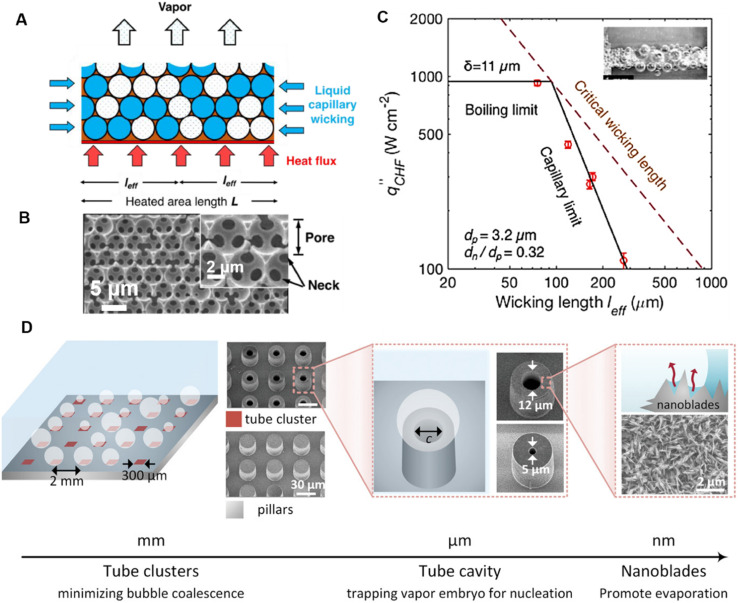
(A) Schematic of liquid and vapor two-phase flow inside the confined cavities of copper inverse opals during capillary-driven boiling.^73^ Lateral liquid delivery over the heated area is driven by capillary pressures of the porous structure during boiling. The length *L* of heated areal footprint determines the liquid wicking length *l*_eff_ under capillary pressures, and the porous wick thickness *δ* decides the vapor transport distance. Reproduced with permission from ref. 73. Copyright 2021 Elsevier. (B) Top view scanning electron microscopy (SEM) images of the well-ordered inverse opal capillary structure.^73^ Reproduced with permission from ref. 73. Copyright 2021 Elsevier. (C) Effect of liquid wicking length on the critical heat flux of capillary-driven boiling from inverse opals samples having 3.2 μm pore diameter.^73^ The boiling CHF decreases after the liquid wicking length increases beyond a critical value, suggesting a transition from the boiling limit regime to the capillary limit regime. *d*_p_ refers to pore diameter while *d*_n_ refers to neck diameter. Reproduced with permission from ref. 73. Copyright 2021 Elsevier. (D) Schematics and SEM images of a hierarchically structured TIP surface (h-TIP) that exhibits capillary wicking while controlling vapor nucleation using multiple length scales.^72^ Reproduced with permission from ref. 72. Copyright 2022 Wiley.

Apart from increasing nucleation site density, micro/nanocavities also induce capillary force on the surrounding liquid which facilitates the timely replenishment of liquid to its surface. Hence, the capillary wicking mechanism has shown the potential to significantly increase CHF.^60^ To improve heat transfer performance and delay CHF, the micro/nanostructure morphology and length scale play a pivotal role to optimize the interplay between capillary pressure and permeability.^61^ Innovative designs such as porous biomimetic structures,^62^ metal organic framework-based surfaces,^63^ and fractal surfaces^64,65^ have all been shown to demonstrate significant CHF enhancements due to enhanced capillary action. Enhanced capillary action leading to increased bubble departure frequency can also improve performance.^66^ To investigate capillary-action attributed enhancements in detail, well-ordered microporous wick structures were designed and investigated (Fig. 2A and B).^67^ CHF in these porous wicks depends on the capillary limit, defined as the ability of the liquid to wet the surface, as well as the boiling limit, defined as the ability of the liquid to rewet the surface after vapor extraction. While a reduction in wicking length leads to enhancement in CHF in the capillary limit, reduction below a certain limit can lead to transition to the boiling limit where CHF enhancements were no longer observed (Fig. 2C). Similar transition effects are also observed on increasing structure thickness beyond an optimum wick thickness. While numerous boiling CHF models for unstructured surfaces have been developed,^68–70^ similar models for structured surfaces remain scarce. A unified model combining the effects of surface roughness and wicking has been shown to give good results and can be utilized to guide design.^71^

Extreme boiling enhancements can be realized by combining the abovementioned enhancement mechanisms of increased nucleation sites, optimum cavity geometry and capillary wicking.^72^ Heat transfer coefficient enhancements of 389% and CHF enhancements of 138% have been recently reported on a three-tier hierarchical tube-cluster in pillar (h-TIP) surface. This strategy enables increased nucleation *via* micron-scale cavities, enhanced capillary action/evaporation through nanostructures and minimal bubble coalescence *via* well-spaced tube clusters (Fig. 2D). The tube cluster spacing approach counters the issue of increased bubble coalescence typically seen on surfaces with increased nucleation sites, leading to simultaneous enhancements in CHF and HTC.

### 2.2 Effect of surface wettability and fluid surface tension on pool boiling

**Fig. 3:**
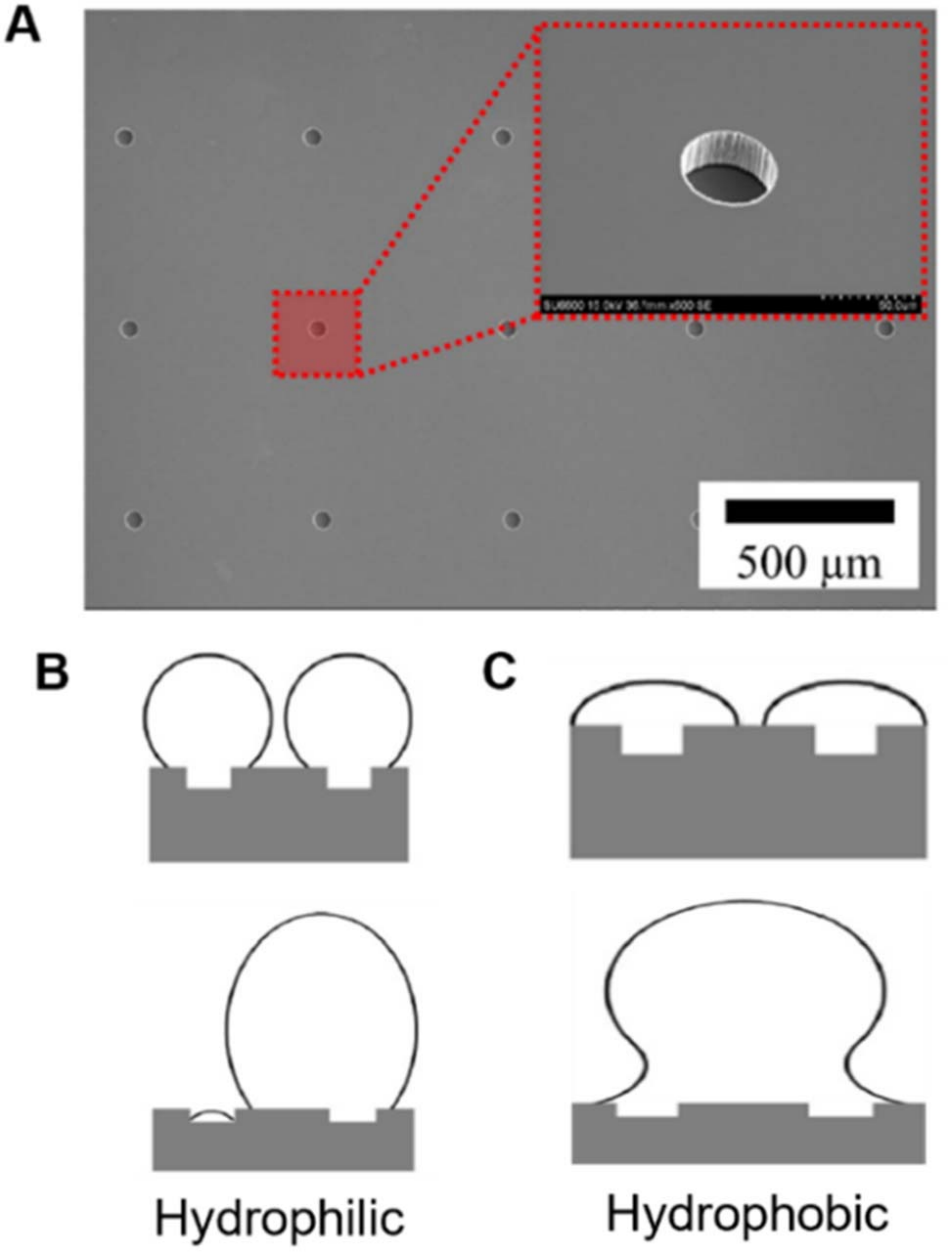
(A) SEM images of micro-hole patterned surface.^67^ Bubble nucleation and growth on microhole patterned (B) hydrophilic^67^ and (C) hydrophobic surfaces at low wall superheats.^67^ Reproduced with permission from ref. 67. Copyright 2020 Elsevier.

The effect of surface wettability on pool boiling performance has been widely examined.^74–76^ The nucleation rate during pool boiling is significantly dependent on surface wettability.^77^ Hydrophobic surfaces exhibit higher bubble nucleation sites due to the presence of the non-wetting spots, enabling the entrapment of vapor embryo.^78^ The effect of altering surface wettability to produce superhydrophilic surfaces (*via* structured surfaces) on boiling performance with water as the working fluid has been another focus area gaining traction past few years in terms of experimental studies.^79^ Nickel inverse-opal structures transitioning from hydrophilic before boiling to hydrophobic within a few minutes after boiling showcases an example where altered wettability is naturally achieved.^52^ Artificial cavities on a silicon surface having a Teflon coating applied to impart hydrophobicity have shown differing bubble coalescence characteristics for hydrophilic and hydrophobic surfaces (Fig. 3A).^67^ Hydrophilic surfaces primarily demonstrate horizontal bubble coalescence while hydrophobic surfaces show vertical bubble coalescence (Fig. 3B and C). Hydrophobic surfaces were found to perform better due to increased departure frequency caused by increased bubble coalescence. Residual vapor left behind on the hydrophobic surface after bubble coalescence leads to a reduction in waiting time for the next bubble nucleation event, thereby enhancing heat transfer. Superhydrophobic surfaces can also enable nucleation at lower wall superheat when compared to superhydrophilic surfaces.^80^ However, vapor agglomeration resulting from bubble coalescence and larger surface tension forces preventing bubble detachment on superhydrophobic surfaces can also hinder heat transfer at moderate and high heat flux when compared to superhydrophilic surfaces and thus, care needs to be taken while utilizing such substrates.

Pool boiling enhancements with low-surface tension working fluids, such as dielectrics and refrigerants, have been demonstrated *via* structured surfaces such as copper spherical granules,^1^ silicon dioxide thin film nanocoated surfaces^81^ and zinc-oxide nanowires.^82^ Heat transfer coefficient enhancements up to 200% demonstrated with commercial nanoFLUX coatings utilizing R-134a and R-245fa have primarily been attributed to increased nucleation sites, increased capillary pumping, and enhanced evaporation of wicked liquid beneath growing vapor bubbles.^83^ The interesting phenomena of decreased wall superheat on increasing heat flux, termed ‘hookback’, has been shown with FC-72.^84^ This is caused due to simultaneous activation of numerous submicron cavities and has been found to be dependent on the level of subcooling and the size-range of cavities. Since capillary wicking for low-surface tension fluids is lower than high surface-tension fluids such as water, the role of pre-existing liquid also plays an important role in addition to capillary wicking for CHF enhancement with low surface tension working fluids.^85^

### 2.3 Pool boiling visualization techniques

**Fig. 4:**
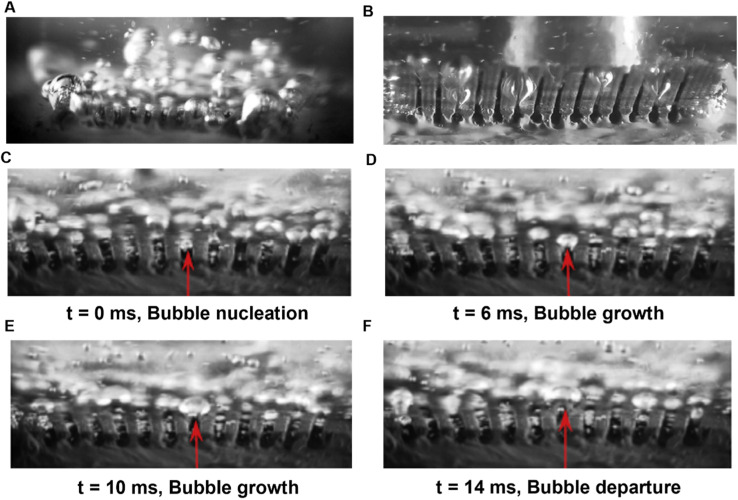
Optical photograph demonstrating more nucleation for (A) porous structured reentrant cavity surface^88^ when compared against (B) non-porous reentrant cavity surface with water as the working fluid.^88^ (C–F) Bubble nucleation, growth and departure captured for porous reentrant cavity surface with ethanol as the working fluid.^88^ Reproduced with permission from ref. 88. Copyright 2016 Elsevier.

Observation of bubble growth and departure are critical in furthering our understanding of fundamental boiling characteristics and bubble dynamics. Most studies thus far have employed high-speed camera imaging to determine fundamental parameters of importance for boiling.^86,87^ Reentrant cavity surfaces have received significant attention in the past and have been shown to induce earlier onset of nucleate boiling when combined with porous surfaces (Fig. 4A and B). These surfaces have been shown to increase heat transfer coefficients up to 500% at low and moderate heat flux conditions due to an increase in nucleation sites and rewetting.^88,89^ Imaging outside the working tool has enabled characterization of the bubble ebullition cycle with bubble nucleation, growth and departure being captured (Fig. 4C–F). Effects of subcooling on bubble nucleation have been demonstrated with a graphene oxide nanocoating on plain copper surface.^90^ Smaller bubble departure diameters on increasing subcooling led to delays in bubble coalescence and thereby increased critical heat flux. While such visualization techniques have contributed significantly to boiling studies, they only provide far-field visualization of the boiling phenomenon. Due to resolution limitations, these techniques are unable to reveal details of the bubble evolution and interaction at the three-phase contact line during microscopic level examination. Recent studies have utilized high resolution techniques to overcome these limitations. Infrared imaging and endoscopic visualization are two such visualization techniques that have gained traction over the past few years, and we focus our attention on these two techniques.

**Fig. 5:**
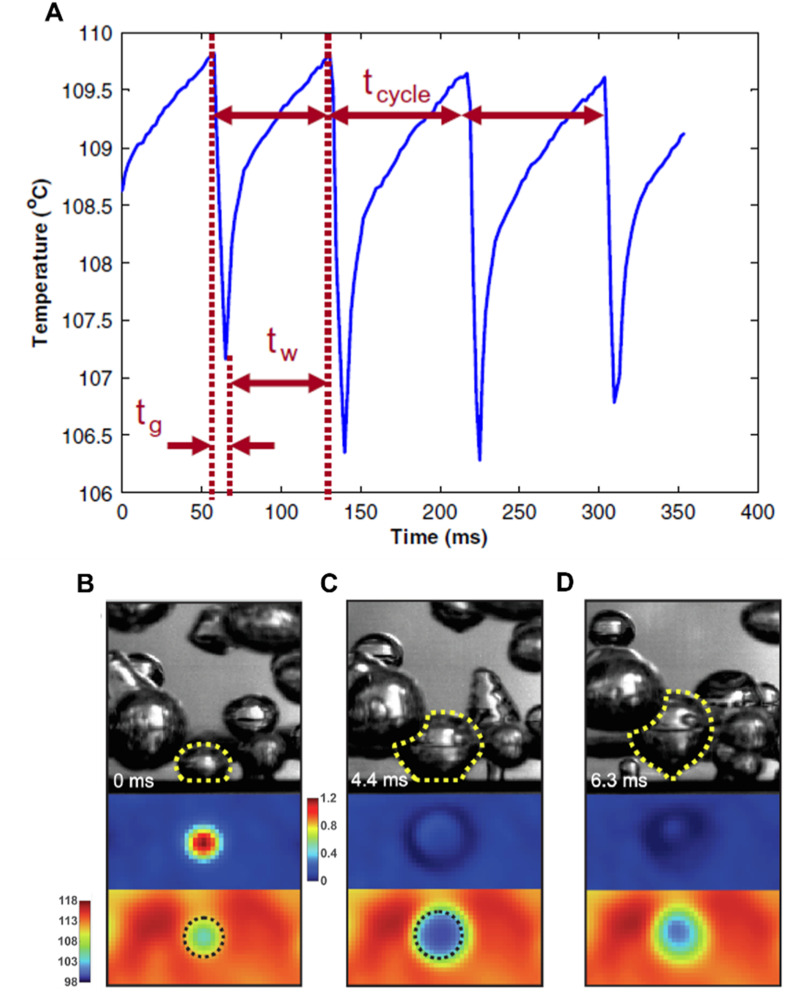
(A) Temperature distribution over time below a nucleation site obtained through IR thermometry.^103^*t*_g_ refers to bubble growth time and *t*_w_ refers to wait time. Reproduced with permission from ref. 103. Copyright 2010 Elsevier. Bubble ebullition cycle displaying (B) nucleation,^107^ (C) growth,^107^ and (D) departure^107^ captured with synchronized high-speed camera and IR camera. Reproduced with permission from ref. 107. Copyright 2022 Elsevier.

Infrared (IR) imaging techniques have been utilized in the wall heat flux partitioning approach to determine local heat flux during pool boiling. While empirical correlations for determination of heat flux have been widely used in the past,^91–95^ the partitioning approach has also been shown to perform well.^96^ The local heat flux in pool boiling has generally been understood to be composed of three parts: (1) convective heat flux, (2) quenching/transient conduction heat flux, and (3) evaporative heat flux. IR imaging can enable extraction of bubble parameters such as size, frequency, nucleation site density, waiting time and these parameters can then be incorporated into the heat flux partitioning model.^97^ Inclusion of parameters such as roughness ratio to account for the area increase in microstructured surfaces have been demonstrated to give good predictions for structured substrates as well.^98^ Similar heat flux partitioning models have also been developed for subcooled and nucleate flow boiling.^99–101^ Microlayer temperature distribution has also been obtained with IR thermography.^102^ High-speed imaging and IR thermometry have been used in conjunction to estimate bubble parameters with observations of bubble radius, microlayer radius and dry out radius showing good agreement with prior models.^103^ The characteristic temperature response under a growing bubble which then departs has also been reported (Fig. 5A),^104^ showcasing the slow heating and rapid cooling process before and after bubble departure. Similar IR imaging techniques have also been applied with nanofluids, where a reduction in bubble departure frequency and nucleation site density led to a 50% reduction in HTC when compared to water.^105,106^ IR imaging has been utilized to determine temperature distributions during boiling on a 50 μm thick zirconium foil (Fig. 5B–D).^107^ The importance of substrate thickness during boiling has been reported in this study with the absence of dry spot formation at bubble base for thin metallic foils, which contrasts with the behavior seen for substrates with larger thickness. This leads to lower evaporative heat flux contribution with similar convective and quenching contributions to the local heat flux. A combination of particle image velocimetry and IR thermometry has recently shown possible links between fluid flow and heat transfer during pool boiling.^108^ Two color laser induced fluorescence was used to track fluid flow while IR thermometry was utilized to obtain temperature distribution. Results showed formation of vortices on the sides of rising bubbles led to mixing close to the heater with IR imaging showing lower temperatures at nucleation sites.^109^ IR imaging has also been utilized to detect liquid/vapor phases in contact with the substrate during boiling and for determination of the wetted are fraction.^110^ This methodology relies on the difference in IR intensity with higher intensity being observed for liquid regions and lower intensity for bubble regions.^111^

**Fig. 6:**
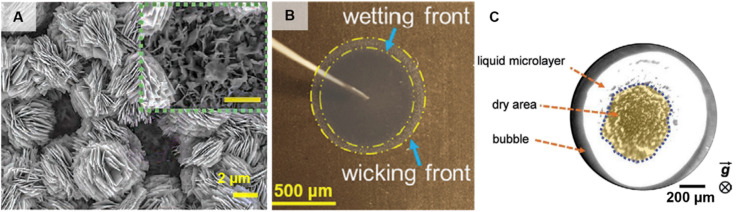
(A) SEM images of a CuO structure having nanoflower morphology.^112^ (B) An optical photograph of a deionized water droplet wicking on the nanoflower structured surface where dotted yellow lines delineate the wicking and wetting fronts and gravity points into the page.^112^ (C) Endoscopic photograph of the bubble base on the nanoflower-structured surface as seen during boiling having no wicking capability after 375 days of exposure to air.^112^ Reproduced with permission from ref. 112. Copyright 2022 Wiley.

Recently, a new technique of utilizing endoscopic visualization inside the work pool has overcome the existing limitation of far-field visualization. Hierarchical copper structures (Fig. 6A) having high wickability were shown to demonstrate enhancements over a period of 1 year, thus demonstrating durability.^112^ Endoscope characterization showed the presence of retained liquid film in addition to the microlayer for the hierarchical surface (Fig. 6B). This retained film is gradually replaced by the dry area after 375 days, after which the surface loses it wicking potential, signified by disappearance of wicking front (Fig. 6C) due to adsorption of hydrophobic volatile organic compounds (VOCs) from air. Endoscopic observations have also enabled definition of a new dimensionless number, retention no. (Ret), which is the ratio of evaporated mass flux of liquid retained within structures to mass flux of vapor leaving the surface due to complete evaporation.^113^ The CHF is found to vary linearly with Ret and experiments performed with surfaces having similar wickability but different water content showed Ret being a better predictor of CHF than wickability. The proposed retention number based CHF model is particularly important for structured surfaces without any interconnected pores where the effects of wickability are negligible. Such endoscopic visualization studies have also aided in determining a linear relationship between bubble departure diameter and CHF^114^ and identification of a wicking area between the dry spot and microlayer region in novel metallic microchannels.^115^

To understand fundamental physical phenomena driving boiling performance, many other innovative methods have also been demonstrated in recent studies. One such example is the usage of temperature sensitive paint to measure wall temperature distribution.^116^ This technique enables simultaneous measurement of both wall temperature distribution and gas–liquid interface, and thus could potentially be used in future studies as well. Additionally, MEMS sensors have also been widely employed for surface temperature measurement due to high-resolution capabilities.^30,36,117,118^ Thus, technological advancements have facilitated development of a wide variety of high-resolution techniques and future boiling studies can be performed in conjunction with such devices instead of solely relying on traditional thermocouple/RTD measurements.

## 3. Flow boiling

**Fig. 7:**
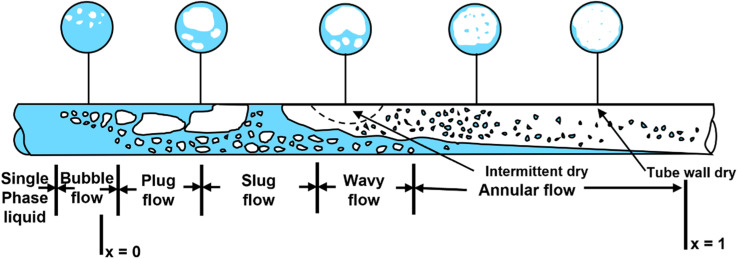
Subcooled-inlet flow boiling patterns as a function of vapor quality (*x*) showing bubbly, plug, slug, wavy and annular flow followed by dry-out.^120^

The mechanisms governing flow boiling heat transfer are more complex than pool boiling due to the increased importance of forces such as buoyancy and inertia. Inertia, surface tension, shear, buoyancy, and evaporation momentum forces are significant for flow boiling with varying relative values for these forces for microchannels and macrochannels.^119^Fig. 7 depicts the flow boiling process with various flow regimes for subcooled-inlet conditions. Single-phase liquid enters the tube and upon incrementing heat flux values, the wall superheat reaches a sufficiently high value for activation of nucleation sites and onset of nucleate boiling. At low vapor qualities, bubbly, slug, and stratified flow are typically observed while annular flow is observed at high vapor qualities. On increasing the heat flux to a sufficiently high value, partial dry-out of the tube wall begins followed by complete dry-out characterized by significantly higher wall temperatures.

Flow boiling heat transfer has primarily been categorized as nucleate boiling dominant or convective boiling dominant depending on the relative effects of heat flux and mass flux. Increased effects of heat flux on the heat transfer coefficient with negligible mass flux effects have been indicative of nucleate boiling dominance,^121–125^ while increased effects of mass flux and negligible effects of heat flux on heat transfer coefficients are considered to signify convective boiling dominance.^126–128^ Heat transfer coefficients typically decrease as the vapor quality increases in nucleate boiling dominant regimes due to suppression of bubbly and slug flow along the tube length. On the other hand, convective boiling dominance results in the heat transfer coefficient increasing across vapor qualities due to enhanced evaporation from the progressively thinner liquid film surrounding tube wall.^129^ Previous studies have also shown nucleate and convective boiling effects to co-exist with neither mechanism demonstrating dominance.^130–133^ These relative effects of nucleation and convection have also formed the basis for a vast majority of flow boiling correlations to predict heat transfer coefficients. Three broad categories for correlations can be defined as follows: (1) superposition models, (2) asymptotic models, and (3) statistical models. Superposition models consist of two terms with each term quantifying the relative weight of nucleation and convection in the flow boiling process.^94,134–136^ The nucleation term consists of a nucleate pool boiling heat transfer coefficient correlation and a suppression factor to account for flow boiling. The convection term consists of a single-phase heat transfer coefficient and an enhancement factor to account for two-phase flow. Asymptotic models select one of the two mechanisms based on their absolute values since the heat transfer coefficient is considered to approach nucleate or convective boiling dominance,^137,138^ while statistical models rely on fitting experimental data to a range of non-dimensional numbers.^121,123,130,139^ The importance of vapor quality has also been highlighted in a correlation proposed by Lee and Mudawar^140^ with nucleate boiling dominating at low vapor qualities (*x* < 0.05) and convective boiling dominating at higher vapor qualities (*x* > 0.05).

In addition to empirical correlations, physics-based models have also been proposed for heat transfer coefficient prediction. While such mechanistic models are far fewer in number (when compared to empirical correlations) due to the complex nature of flow boiling, they reveal important physical characteristics not captured by correlations. Jacobi and Thome^141^ proposed a two-zone heat transfer model (liquid slug and elongated vapor bubble) for microchannels with film evaporation being considered the dominant mechanism. Thome *et al.*^142^ extended on this work with a three-zone model where a vapor slug was added to the two earlier proposed zones and a cyclic passage of liquid slug, elongated bubble and vapor slug were considered to predict local heat transfer coefficient. An updated version of the three-zone model has recently been proposed by Magnini *et al.*^143^ with liquid film thickness being determined by accounting for bubble proximity effects and bubble nose velocity being calculated by capillary flow theory. Qu and Mudawar^144^ proposed a model to determine the liquid film thickness by considering liquid droplet entrainment in the vapor core and the heat transfer coefficient for microchannels was then determined by dividing the liquid thermal conductivity and film thickness.

### 3.1 Nucleation sites, turbulence, and instability suppression

Flow boiling heat transfer mechanisms are governed by two distinct phenomena: (1) nucleate boiling contribution attributed to bubble growth and departure at low vapor qualities and (2) convective boiling contribution attributed to liquid film evaporation at intermediate-high vapor qualities.^129^ Heat transfer characteristics are highly dependent on surface morphology and can lead to efficient heat transfer by tuning nucleation site geometry and pore density.^145^ Graphene based nanocomposite coatings on copper substrates have proven to be an effective method to increase pore density, thereby increasing nucleate boiling contribution.^146^ Variation of coating concentration can increase pore density and further improve thermal performance. Similar effects of increased pore density (caused due to increased current density) leading to enhancements have been observed with Cu–Al_2_O_3_ nanocomposite coatings on copper^147^ and Cu–TiO_2_ micro/nanostructured surfaces.^148^ A reduction in static contact angle with increased porosity resulting in enhanced bubble departure frequency can give further enhancements.

An important consideration when considering utilization of coatings for performance enhancements is the effect of coating thermal conductivity on overall performance. Coatings with high thermal conductivity can improve performance.^146^ Interconnected pores formed through structure fabrication also leads to enhanced wicking^149,150^ and can increase film evaporation contributions as well as bubble departure frequency. A laser-induced fluorescence technique with 1 μm diameter tracer particles has been used to characterize liquid velocity inside pores.^151^ Like pool boiling, the presence of numerous nucleation cavities also facilitate lower wall superheat ONB when compared to unstructured surfaces.^152^ Higher HTC values at low mass fluxes have been attributed to enhanced nucleation activity and film evaporation with microporous copper surfaces.^153^ Furthermore, porous inserts have shown enhancements at low heat flux values where the effects of convective boiling are more dominant without showing any significant enhancements at high heat flux values.^154^ This underlines the importance of structure characteristics and the need to perform detailed characterization studies to distinguish between various enhancement mechanisms.

Wall structures can also lead to disruptions in the boundary layer and cause perturbations in the liquid–vapor interface, promoting mixing and thereby enhancing heat transfer.^155^ Flow boiling instabilities often occur in mini/microchannel heat sinks and are generally characterized by increased pressure drop and wall temperature fluctuations.^156^ Dynamic instabilities such as bubble clogging in confined spaces can lead to flow reversal in microchannels. Onset of such instabilities during flow boiling are dependent on parameters such as channel geometry and operating conditions with larger inlet subcooling often associated with faster occurrence of this phenomenon.^157–159^ Vapor generation in microchannels has been shown to cause pressure drop fluctuations in rectangular parallel microchannels as well.^160–163^ Thus, flow boiling instabilities in microchannels can hurt enhancements that would otherwise be expected by creation of micro/nanoscale features on the base substrate and needs to be factored in while reporting results for better comparison between studies. Recent work has aimed at reduction of such instabilities with a bi-porous heat sink design sintered with copper woven tape being shown to reduce pressure drop fluctuations.^164^ An innovative 3D manifold microchannel design integrated with silicon nanowires has been shown to reduce these fluctuations considerably.^165^ While the top manifold enables vapor escape, the bottom microchannel exhibits enhanced thin film evaporation *via* enhanced capillarity through interconnected nanocavities. In addition, surface wetting shifts from being hydrophilic before tests to hydrophobic after tests leading to degradation in performance at high mass flux/heat flux operating conditions where capillarity would otherwise have led to enhancements. While pressure oscillations are almost always accompanied by deterioration in HTCs, a recent study has reported heat transfer enhancements utilizing a copper wire mesh screen.^166^ Peak pressure fluctuations coincided with peak HTC enhancements and bubble burst caused by intense bubble activity was observed with nucleate boiling dominance for all heat flux values tested.

Herringbone microfins have shown to improve heat transfer performance during flow boiling. Annulus herringbone HTC enhancements up to 2.5 times of a plain surface have been made possible largely due to enhanced turbulence.^167,168^ Similar enhancements have also been shown for enhanced structures inside tubing with refrigerants as the working fluid.^169^ The presence of additional nucleation sites and gas-phase shear resulting in enhanced mixing led to performance improvements. Higher HTC values at higher mass flux are caused due to a thinner liquid film surrounding the tube wall, leading to lower conduction resistance and enhanced film evaporation.

Hysteresis effects have been reported for hydrophilic surfaces where the HTC is not found to be the same when going from lower vapor quality to higher vapor quality and *vice versa*. An approximately 20% increase in HTC at low mass flux values was seen in carbon fiber reinforced matrix with R-245fa when tests progressed from higher to lower vapor quality due to vapor filled nucleation cavities getting activated when liquid rewets.^170^

**Fig. 8:**
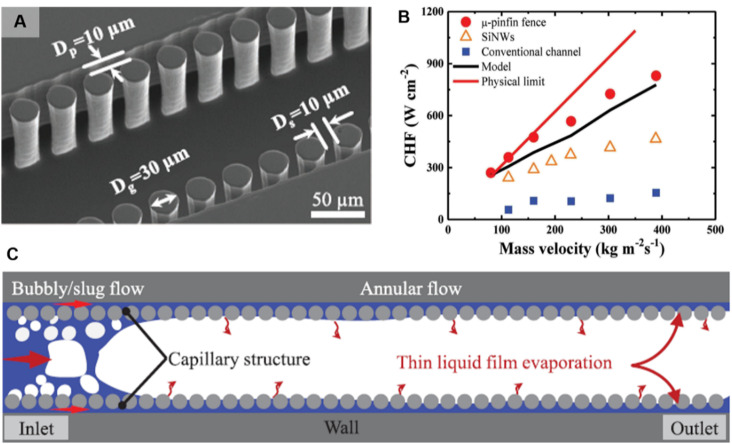
(A) SEM image of a microchannel decorated with a μ-pinfin fence.^164^*D*_p_ and *D*_s_ refer to diameter and spacing of the μ-pinfin arrays while *D*_g_ refers to the gap between the μ-pinfin arrays and sidewall. (B) Plot showing the variation of CHFs under mass velocity and an achievement of a high CHF of 830 W cm^−2^ in the μ-pinfin fence at a mass velocity of 396 kg m^−2^ s^−1^, 437% higher than conventional two-phase flow.^164^ (C) Schematic showing the full separation in two-phase flow using the μ-pinfin fence, which is denoted by the silver circles.^164^ Reproduced with permission from ref. 164. Copyright 2020 Wiley.

Recent work incorporating micro-pin fin fences aimed at rectifying chaotic flow regimes in silicon microchannel flow boiling has enabled efficient heat transfer performance.^164^ These micro-pin fences are designed along the sidewalls with geometry parameters that enable wavelengths lower than the Kelvin–Helmholtz (KH) instability number (Fig. 8A). Presence of a single flow regime – stable annular flow – leads to highly efficient thin film evaporation that results in heat transfer performance close to the physical limit of boiling (Fig. 8B and C). The superhydrophilic fences avoid the issue of entrained droplets within the vapor core and have also been shown to work with a low-surface tension fluid (HFE-7100), where such enhancements are much more difficult to obtain due to the lower KH number. Micro-pin fin fences also demonstrate lower wall temperature fluctuation due to the stable annular flow.^171^ Building on this work, silicon nanowire pin fin fences enable even better performance when compared to micro-pin fin fences due to higher capillary pressure.^172^ This is seen in the exit vapor quality being 15% higher than that of micro-pin fin fence boiling for constant temperature boundary conditions. Furthermore, a 43% reduction in pressure drop was also been reported due to enhanced rewetting. Microporous decorated sidewalls also rely on the mechanism of liquid–vapor separation for flow boiling enhancements and show an exit vapor quality of 0.3 compared to 0.1 for plain wall microchannels, signifying more efficient boiling.^173^

**Fig. 9:**
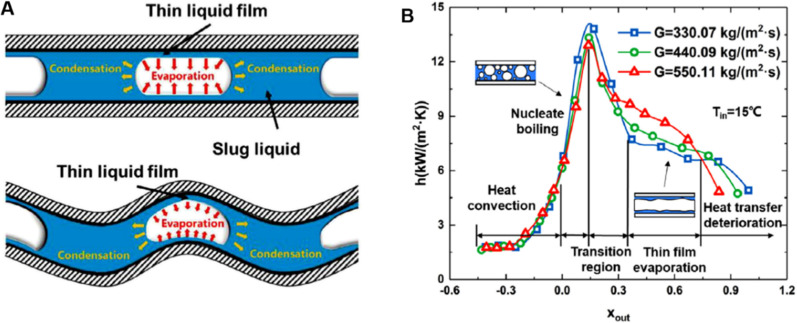
(A) Top views of confined bubbles in wavy and straight microchannels.^179^ (B) Variation of flow boiling HTC (*h*) as a function of outlet vapor quality (*x*_out_) for different mass fluxes (*G*) at an inlet saturation temperature (*T*_in_) of 15 °C.^179^ Reproduced with permission from ref. 179. Copyright 2021 Elsevier.

Research with dielectric fluids as the working fluid in microchannels has gained prominence due to their usage in electronics cooling applications.^174^ Additive manufacturing techniques such as Selective Laser Melting (SLM) have been employed to fabricate 3D porous metallic structures with FC-72 as the working fluid.^175^ Fractal hydrophilic networks have also demonstrated up to 82% CHF enhancement due to enhanced wetting through networks.^176^ Application of piranha pin fins with HFE-7000 have demonstrated ultra-high cooling capabilities as well.^177^ While PDMS based microchannels can be a good alternative to conventional silicon microchannels for flexible electronic systems, poor thermophysical properties associated with such devices lead to less than satisfactory heat transfer performance. To overcome this limitation, PDMS wick structures with separate vapor removal pathways have been designed.^178^ Such micropillars enable high capillary pressure and high permeability enabling stable liquid film evaporation characterized by stable wall temperatures at high heat flux operating conditions. Phase separation and improved global liquid supply allow CHFs to approach those observed on silicon and copper microchannel heat sinks. Another innovative design with HFE-7100 as the working fluid involved fabrication of microporous structures in wavy microchannels resulting in ∼60% HTC enhancement and ∼28% CHF enhancement when compared to straight microchannels (Fig. 9A).^179^ Improvements in the nucleate boiling regime are caused due to increased nucleation sites and higher bubble detachment rate driven by centrifugal acceleration, while improvements in the film evaporation regime are due to thinner liquid film facilitated by the wavy channel (Fig. 9B). Heat transfer coefficients are independent of mass flux in the nucleate boiling zone while mass flux plays a role in the thin film evaporation zone. Increased bubble nucleation and growth in the wavy concave region of the microchannel presents opportunities to further enhance HTC by increasing the wavy concave curvature and reducing wavy convex regions. Application of porous coatings in microchannels have also shown enhanced heat transfer coefficients with HFE-7200.^42^ Creation of cavities in the desired range (0.6 to 3 μm) led to enhanced nucleation at low vapor qualities which resulted in peak HTC enhancement at a heat flux where bubbly flow and slug flow were found to dominate. In terms of local heat transfer characteristics, a sharp peak at low vapor qualities was followed by a gradual decline, representative of nucleate boiling suppression. At high vapor qualities, enhanced mixing led to heat transfer coefficient improvements despite nucleate boiling suppression. Early dry out for low mass flux while greater nucleate boiling suppression at highest mass flux led to highest average HTC enhancements at the intermediate mass flux.

### 3.2 Effect of micro/nanostructure wettability on flow boiling

Investigation of surfaces with heterogenous wettability has involved varying spacing between cavities and trials of varying shape patterns.^180–182^ Amongst the shapes investigated, triangular shaped patterns were found to have the highest bubble lifting force, which led to HTCs. Narrow inter-spacing between cavities led to premature bubble coalescence which prevented liquid replenishment and deteriorated heat transfer. Lower ONB superheats for structured surfaces in flow boiling are caused due to presence of additional cavities. The combination of hydrophobic coatings on a hydrophilic substrate has dual advantages: (1) corners formed between these varying wettability surfaces serve as nucleation sites forming nanoscale bubbles and (2) hydrophilic regions supply liquid facilitating bubble detachment.^183^ Superhydrophobic porous copper surfaces have also enabled higher heat transfer coefficients and examination of effects of subcooling have been conducted.^184^ The presence of numerous cavities and interconnected pores helps prevents flooding. Superhydrophobic surfaces also make it difficult for bubbles to depart resulting in virtually no change in flow regime throughout the operating conditions.

### 3.3 Effect of micro/nanostructure wickability on flow boiling

The balance of capillary pressure and permeability has been shown to be of critical importance in wick structure development. When the pressure drop of wicked liquid is greater than the capillary pressure, liquid supply to these structures is limited and deteriorates heat transfer. To overcome this limitation, gradient wick structures are designed integrating both, large pore sizes to enhance permeability and small pore sizes to enhance capillarity.^185^ These gradient wick structures were found to outperform homogenous wick channels, solid fin channels and plain copper surfaces due to the resultant enhanced evaporation. More wicked inflow was observed at higher mass fluxes owing to higher far field pressures leading to higher HTCs. It should be noted however that a high degree of turbulence in annular flow could lead to the unintended consequence of earlier onset of dry-out for structured surfaces and thereby potentially lead to lower averaged heat transfer coefficients across the vapor quality range. This is particularly true for larger diameter tubes where the effects of shear force and gravity dominate over surface tension, unlike the case for mini/microchannels.^119^

### 3.4 Structure durability

While durability issues for micro/nanostructured surfaces pose limitations to their eventual applicability for industrial processes,^186^ few flow boiling studies exist which address this area of concern. Recently, microstructured aluminum surfaces demonstrating up to 270% enhancement have displayed good preliminary durability with the structured surface displaying negligible variation in heat transfer/SEM studies after 28 days.^187^ Overall, more work needs to be done in this regard with long term durability tests focused on variation of operating parameters and channel sizes.

## 4. Condensation

**Fig. 10:**
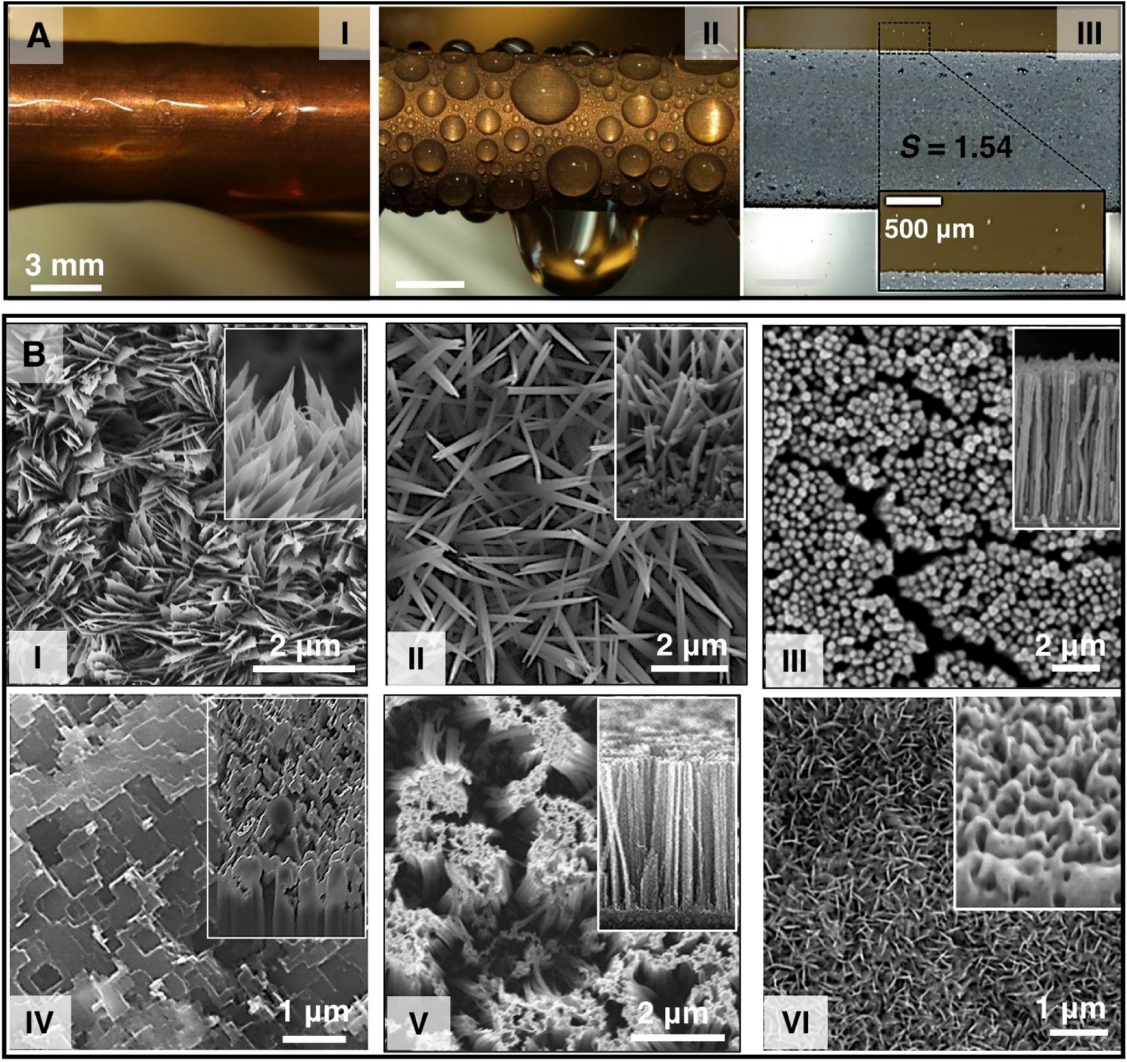
Images showing (A-I) filmwise condensation, (A-II) dropwise condensation, and (A-III) jumping droplet condensation on bare hydrophilic copper surface, hydrophobic copper surface and superhydrophobic CuO nanostructured surfaces respectively.^198^ Reproduced with permission from ref. 198. Copyright 2013 American Chemical Society. Scanning Electron Microscopy (SEM) images of some of the single-tier nanostructured superhydrophobic surfaces which includes (B-I) knife-like copper oxide nanostructures,^198^ (reproduced with permission from ref. 198. Copyright 2013 American Chemical Society) (B-II) copper oxide nanograss,^199^ (reproduced with permission from ref. 199. Copyright 2019 Elsevier) (B-III) copper nanowires,^200^ (reproduced with permission from ref. 200. Copyright 2017 Elsevier) (B-IV) etched aluminum microstructures,^201^ (reproduced with permission from ref. 201. Copyright 2022 Wiley) (B-V) silicon nanowires,^202^ (reproduced with permission from ref. 202. Copyright 2017 Elsevier) (B-VI) aluminum boehmite nanostructures^201^ (reproduced with permission from ref. 201. Copyright 2022 Wiley).

Conventional metallic surfaces suffer from limited heat transfer performance due to condensate accumulation from filmwise condensation (Fig. 10A-I).^18^ The keys to enhancing condensation are prevention of condensate film formation and reduction of condensate droplet departure size.^188^ The condensation droplet nucleation density and rate is dependent on the intrinsic wettability of a surface.^189,190^ Hence, nucleation density is higher on hydrophilic surface compared to hydrophobic surface of lower surface energy.^190^ The nucleation radius of condensate droplets is in the nanometer scale and as a result does not depend on the micro or nanostructures of the surface.^191–193^ Early approaches by researchers to enhance condensation included development of low surface energy promoter coatings to enable dropwise condensation instead of filmwise condensation.^21^ Low surface energy coatings enable dropwise condensation by preventing condensate film formation due to reduced nucleation density combined with efficient continuous droplet shedding (Fig. 10A-II). As a result, dropwise condensation can enable up to an order of magnitude increase in condensation heat transfer.^14^

### 4.1 Rational design of micro and nanostructures for enhanced quiescent condensation

In the last decade there has been increased interest in the development of various micro/nanostructured superhydrophobic surfaces to achieve jumping droplet condensation of steam.^194^ Jumping droplet condensation on superhydrophobic surfaces (Fig. 10A-III) have been reported to enable up to an order of magnitude enhancement in condensation HTC when compared to dropwise condensation on a smooth hydrophobic surface.^18,195^ The larger enhancement of condensation HTC is due to removal of condensate droplets at diameters that are orders of magnitude smaller (<100 μm) when compared to gravity-induced shedding (capillary length, 2.7 mm for water) during dropwise condensation.^196,197^

Recent advancements in superhydrophobic surface development have focused on fabrication techniques for implementing optimized structure capable of preventing condensate flooding at extreme conditions such as very high surface subcooling temperatures, and higher vapor supersaturation conditions (Fig. 10).^18,200,201^ Jumping droplet condensation performance depends largely on the nanostructure shape, size, orientation, and density.^201,203^ For the development of optimized micro and nanostructured surfaces researchers have reported methods such as machining,^204^ sandblasting,^205^ laser ablation,^206^ thermal^207^ or chemical oxidation,^18,208,209^ chemical etching,^210,211^ jet electrolyte micromachining,^212^ photolithography,^205^ nanoimprint lithography,^213^ dry reactive ion etching,^214^ electrodeposition^203^*etc.* However, majority of these micro and nano structure fabrication methods are complex, not scalable, not suitable for large scale industrial application, and are only limited to substrate materials such as silicon which are not commonly used for industrial condenser. Researchers have reported many micro and nanoscale oxide or etched structures that are capable of exhibiting jumping droplet condensation at lower surface subcooling and at low supersaturation conditions. Some of these structures are knifelike nanostructures,^215^ nanograss,^199^ nanowires,^216^ hierarchical-porous nanostructures,^203^ nanocones,^217^ ribbed nanoneedles,^218^ and platelet like nanostructures^201^ fabricated on common substrate materials such as silicon, copper, and aluminum (Fig. 10B). Copper oxide nanostructured surfaces continue to be the mostly widely studied surface mainly due to its simplicity and low cost of fabrication.^215^ A recent review focusing on micro and nanostructures developed solely for condensation applications provides a detailed summary of past studies with relevant performance parameters.^194^

**Fig. 11:**
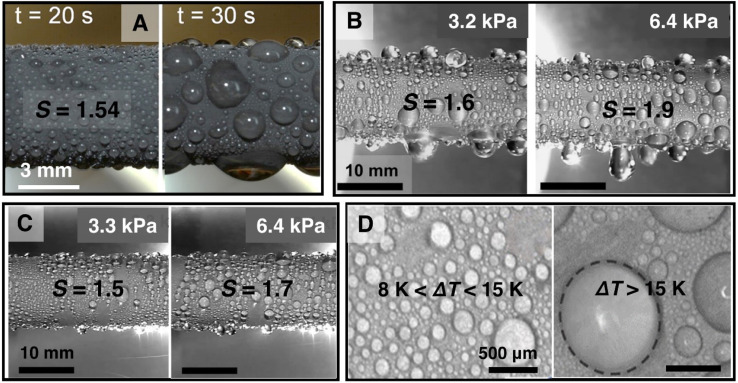
(A) Images showing pinned droplets on a superhydrophobic CuO single-tier nanostructured surface due to condensation induced flooding at high supersaturation condition (*S* = 1.54).^198^ Reproduced with permission from ref. 198. Copyright 2013 American Chemical Society. (B) Images showing pinned droplets on a superhydrophobic single-tier nanostructured aluminum boehmite surface due to condensation induced flooding at high supersaturation conditions *S* = 1.6 and 1.9 for pure vapor pressure *P*_v_ = 3.2 kPa and 6.4 kPa.^201^ Reproduced with permission from ref. 201. Copyright 2022 Wiley. (C) Images showing pinned droplets on a superhydrophobic single-tier microstructured etched aluminum surface due to condensation induced flooding at high supersaturation conditions *S* = 1.5 and 1.5 for pure vapor pressure *P*_v_ = 3.3 kPa and 6.4 kPa.^201^ Reproduced with permission from ref. 201. Copyright 2022 Wiley. (D) Images showing jumping droplet condensation at lower (8 K < Δ*T* < 15 K) and pinned droplets after coalescence at higher (Δ*T* > 15 K) subcooling temperature on a superhydrophobic surface with copper nanowires.^200^ Reproduced with permission from ref. 200. Copyright 2017 Elsevier.

**Fig. 12:**
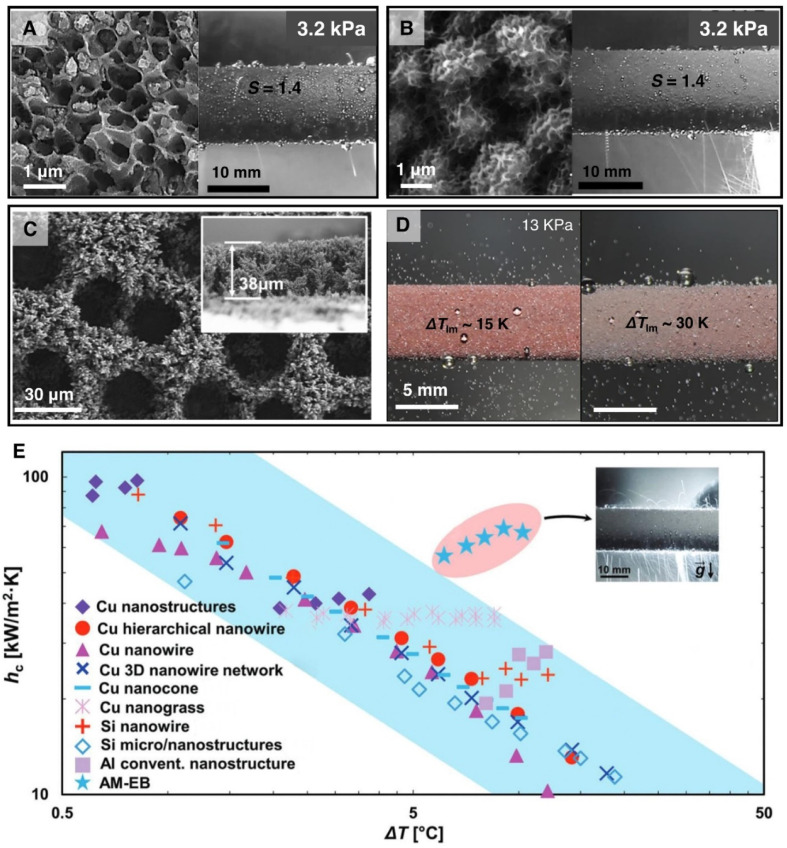
(A) SEM image showing single-tier nanocellular structured superhydrophobic surface which enables jumping droplet condensation at high supersaturation *S* = 1.4 at pure vapor pressure *P*_v_ = 3.2 kPa.^201^ Reproduced with permission from ref. 201. Copyright 2022 Wiley. (B) SEM image of the two-tier nanocellular structured superhydrophobic surface which enables superior jumping droplet condensation at high supersaturation *S* = 1.4 at pure vapor pressure *P*_v_ = 3.2 kPa.^201^ Reproduced with permission from ref. 201. Copyright 2022 Wiley. (C) SEM image showing microscopic morphology of the honeycomb-like electrodeposited copper superhydrophobic surfaces.^203^ Reproduced with permission from ref. 203. Copyright 2021 Elsevier. (D) Images showing jumping droplet condensation on an electrodeposited honeycomb-like porous copper surface at various degrees of surface temperature at steam pressures of 13 kPa.^203^ Reproduced with permission from ref. 203. Copyright 2021 Elsevier. (E) Comparison of the jumping droplet condensation heat transfer coefficient of two-tier cellular nanostructured surface (AM-EB) with other nanostructured superhydrophobic surfaces with respect to subcooling (Δ*T*).^201^ Reproduced with permission from ref. 201. Copyright 2022 Wiley.

Many of the developed surface structures (*i.e.*, single-tier knife-like copper oxide nanostructures, plate like boehmite nanostructures, etched aluminum microstructures, copper nanowires) suffer from interstructure condensation induced flooding at higher subcooling, vapor pressure, and supersaturation condition (*S* = *P*_v_/*P*_sat_) (Fig. 11A–D).^193,201^ Hence, recent studies have focused on the development of rationally designed micro and nanostructures that can prevent such interstructure flooding and exhibit sustainable jumping droplet condensation. For example, studies have reported that fabrication of cellular nanostructures (Fig. 12A) that are capable of confining the condensate droplets within cells preventing lateral spreading induced flooding and as a result can exhibit jumping droplet condensation at higher subcooling and supersaturation condition.^210^ Furthermore, recent studies have shown that addition of multi-tier nanostructures or hierarchical structures on top off such structures can provide anti-flooding superhydrophobic surfaces that can sustain jumping droplet condensation at very high supersaturation conditions (Fig. 12B).^201^ In other work electrodeposition was utilized for fabricating hierarchical honeycomb-like structures on copper surface (Fig. 12C).^203^ The resultant micro and nano structured copper surface exhibited sustainable jumping droplet condition at higher subcooling temperature compared to smooth hydrophobic surfaces (Fig. 12D). Comparison of the jumping droplet condensation HTC of multi-tier cellular nanostructured surface (AM-EB) with previously reported superhydrophobic surfaces *i.e.*, CuO nanostructures,^18^ Cu hierarchical nanostructures,^219^ Cu nanowire,^200^ 3D Cu nanowire,^195^ Cu nanocone,^217^ Cu nanograss,^199^ Si nanowire,^202^ Si micro/nanostructures,^220^ and conventional Al nanostructures,^201^ shows its significantly improved performance at higher subcooling temperatures (Fig. 12E).

**Fig. 13:**
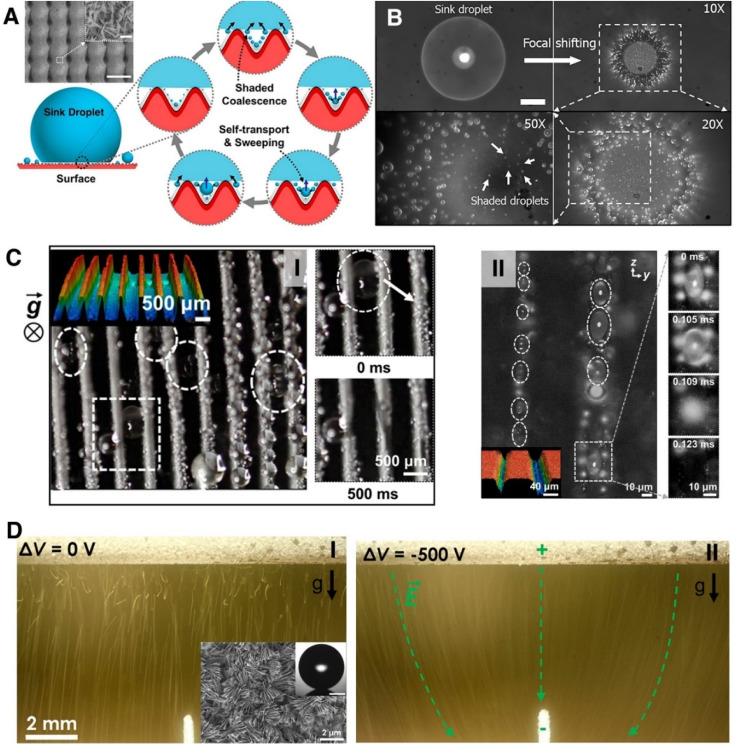
(A) The schematic of shaded coalescence during hierarchical condensation. The large droplet acts as a sink, absorbing droplets surrounding and beneath it. SEM of the hierarchically structured CuO nanoblade surface.^221^ Scale bar: 50 μm. Inset: high-resolution SEM of the CuO nanoblades. Inset scale bar: 1 μm. Reproduced with permission from ref. 221. Copyright 2019 American Chemical Society. (B) Top-view high-speed optical microscopy showing small, shaded droplets surrounding and beneath a large sink CB droplet.^221^ The focal plane in the second (10×), third (50×), and fourth (20×) images is located at the droplet base. Scale bar: 200 μm. Reproduced with permission from ref. 221. Copyright 2019 American Chemical Society. (C-I) Optical microscopy images depicting jumping of a single condensing water droplet out of large-scale (500 μm) superhydrophobic grooves (finned tube).^222^ Reproduced with permission from ref. 222. Copyright 2020 American Chemical Society. (C-II) Images depicting jumping of a single condensing droplet out of small-scale (∼10 μm) grooves.^222^ The squeezed droplets are identified with white dotted circles. Inset image: 3D profile of the grooves. Reproduced with permission from ref. 222. Copyright 2020 American Chemical Society. (D) Long exposure time images showing electric-field-enhanced droplet removal during condensation on a superhydrophobic CuO tube with a copper electrode located beneath it.^226^ (D-I) Electrode at 0 V bias having significant droplet–droplet interactions and return to the surface against gravity. (D-II) Electrode at 500 V bias with no droplet return to the surface and significant attraction of jumping droplets away from the surface. Reproduced with permission from ref. 226. Copyright 2013 ACS.

Recent study has shown that synergistic combination of micro/nanostructured roughness with divergent microcavities of a hierarchically structured surface enables enhanced condensation due to hierarchical condensation mechanism (Fig. 13A).^221^ During hierarchical condensation large Cassie Baxter state droplets suspended on tops of microstructures act as sinks and enables frequent removal of the shaded droplets (Fig. 13A and B). As a result, such structure can prevent progressive flooding, and enable higher overall heat flux than an equivalent jumping-droplet-condensation on single-tier nanostructured surface. Besides droplet coalescence induced jumping droplet on a superhydrophobic surface, researchers have recently demonstrated superhydrophobic-groove-mediated single droplet jumping (Fig. 13C) during condensation and particle-droplet coalescence induced droplet self-transport which can further facilitate condensate droplet removal.^222–225^ Researchers have also demonstrated electric-field-enhanced condensation (Fig. 13D) where it was shown that an external electric field can be applied to prevent the return of the positively charged jumping droplets resulting in a 50% higher overall condensation heat transfer coefficient compared to that on a jumping-droplet surface with no applied field for low supersaturations (<1.12).^226–229^

### 4.2 Low surface tension liquid condensation enhancement

**Fig. 14:**
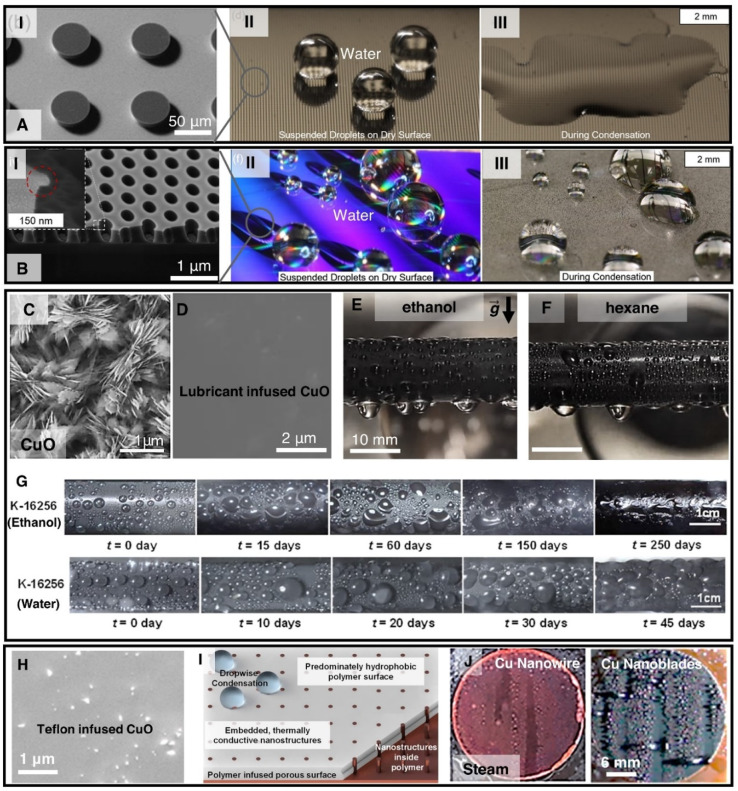
(A-I) Scanning electron microscopy (SEM) image of reentrant pillar surface.^192^ (A-II) Water droplets placed on a reentrant pillar surface. (A-III) Omniphobicity was destroyed during condensation due to transition to the Wenzel state. Reproduced with permission from ref. 192. Copyright 2018 American Chemical Society. (B-I) Scanning electron microscopy (SEM) image of non-wicking reentrant nanoscale cavity surface.^192^ (B-II) Water droplets placed on non-wicking reentrant nanoscale cavity surface. (B-III) Omniphobicity was sustained during condensation due to prevention of Wenzel state propagation. Reproduced with permission from ref. 192. Copyright 2018 American Chemical Society. SEM images of a (C) superhydrophobic CuO nanostructured surface and (D) lubricant infused CuO LIS.^171^ Reproduced with permission from ref. 171. Copyright 2022 American Chemical Society. Images showing dropwise condensation of (E) ethanol condensation at *P*_sat_ of 5.2 kPa and (F) hexane condensation at *P*_sat_ of 10.8 kPa on horizontally oriented CuO lubricant infused tubes.^240^ Reproduced with permission from ref. 240. Copyright 2021 Elsevier. (G) Time-lapse optical images showing ethanol and steam condensation durability of Krytox 16256 infused CuO LIS for up to 250 days and 45 days respectively.^171^ Reproduced with permission from ref. 171. Copyright 2022 American Chemical Society. (H) SEM image showing a polymer infused porous surface (PIPS) consisting of Teflon-AF infused CuO nanostructures. (I) Schematic illustrating the 3D schematic of the fabrication process for PIPS, where the nanopillar structures are infused with polymer, enabling dropwise condensation of liquid.^249^ (J) Images showing dropwise condensation of steam on PIPS made from Cu-nanowires and Cu-nanoblades.^249^ Reproduced with permission from ref. 249. Copyright 2020 American Chemical Society.

In case of low surface tension liquid condensation, regular micro and nanostructured superhydrophobic or omniphobic surfaces suffer from interstructure condensation induced flooding.^192,230^ Studies have shown that it is possible to create reentrant structure geometry that can maintain air pockets within structures, exhibit omniphobicity without any chemical modification by suspending the liquid on top of the structures at Cassie wetting state, and can repel low surface tension liquids (Fig. 14A).^231,232^ However, researchers have experimentally shown that nucleating condensate droplets of any liquid can condense within the reentrant microstructures resulting in condensate induced flooding (Fig. 14A-III).^192^ Furthermore, super-repellant doubly reentrant structured surface which can repel extremely-low-energy liquids such as fluorinated solvents (*i.e.*,FC-72) are unable to withstand condensate induced flooding as it has no defense against condensate nucleating inside the cavities.^233^ Recent study has shown that condensation-resistant omniphobic structured surface is required to have a reentrant cavity structure which can prevent Wenzel state condensate droplets from propagating and the pitch of the structure should be less than the nucleation spacing to prevent nucleation from occurring within every cavity of the surface (Fig. 14B).^192^ However, fabrication of such reentrant structures is highly expensive, not scalable and not practical for large scale condenser application. Furthermore, long term sustainable condensation of low surface tension liquids on reentrant cavity structured surfaces are yet to be reported. Very few surface modification techniques have succeeded in achieving scalable and sustainable dropwise condensation of low surface tension liquids.^234–236^ Studies on lubricant infused structured surfaces (LIS) have introduced the methodology of modifying micro and nanostructured surfaces with lubricant infusion for enabling enhanced condensation of liquids.^230,237–239^ Lubricant infused nanostructured surfaces have successfully been implemented to achieve stable dropwise condensation of toluene, ethanol, hexane, and pentane which are low surface tension fluids (Fig. 14C–F).^234,240–242^ The studies showed that LIS enhances HTC by 200%, 200%, and 450% for condensation of ethanol, hexane, and toluene, respectively when compared to filmwise condensation on an untreated surface. Researchers have shown it is possible to further reduce the condensate droplet departure size and increase the condensation rate on a LIS by introducing vibrational actuation.^243^ However, LISs have limited lifespan depending on choice of lubricant, lubricant condensate miscibility, condensate cloaking by lubricant, and lubricant drainage over time.^244–247^ Until now, the longest reported lifetime of LIS is more than 8 months of continuous ethanol condensation and 45 days for water condensation (Fig. 14G) for CuO nanostructured surfaces infused with high viscosity Krytox 16256 lubricant (5216 mPa s)^171^ before transitioning to filmwise condensation due to lubricant depletion. To alleviate the limitation of LIS due to oil depletion during condensation applications recent studies have proposed complex and expensive mechanisms *i.e.*, lubricant replenishment through brushing of the surface.^248^ Due to these limitations of LIS, to achieve dropwise condensation of low surface tension liquids researchers have resorted to implementation of low surface energy and low hysteresis coating *i.e.*, iCVD and polydimethylsiloxane-silane coated smooth surfaces.^235,236^

Recently researchers have also investigated hydrophobic polymer (Teflon AF) infused nanostructured porous surface for condensation application, known as PIPS (Fig. 14H–J). The PIPS was shown to be significantly more durable than a Teflon coated surface, delivering 700% improvement over an uncoated surface for 200 days.^249^ However, PIPS have only been studied for condensation of steam (Fig. 14J) and its potential for low surface tension liquids condensation is yet to be investigated.

### 4.3 Hybrid wettability surfaces for enhanced dropwise condensation

Recent advancements have also focused on development of nature inspired biphilic or hybrid wettability surfaces which consist of a combination of different wettability regions (*i.e.*, superhydrophilic, hydrophilic, hydrophobic, and superhydrophobic).^250^ Hybrid wettability surfaces have been shown to enhance condensation due to increased preferential condensate nucleation at the hydrophilic regions as condensate nucleation on hydrophobic surfaces requires a higher degree of supersaturation.^191,251^ Furthermore, unlike on a completely hydrophilic surface, the presence of the hydrophobic region a hybrid surface can enhance droplet shedding and prevents condensate flooding at higher subcooling temperatures.^250,252^

**Fig. 15:**
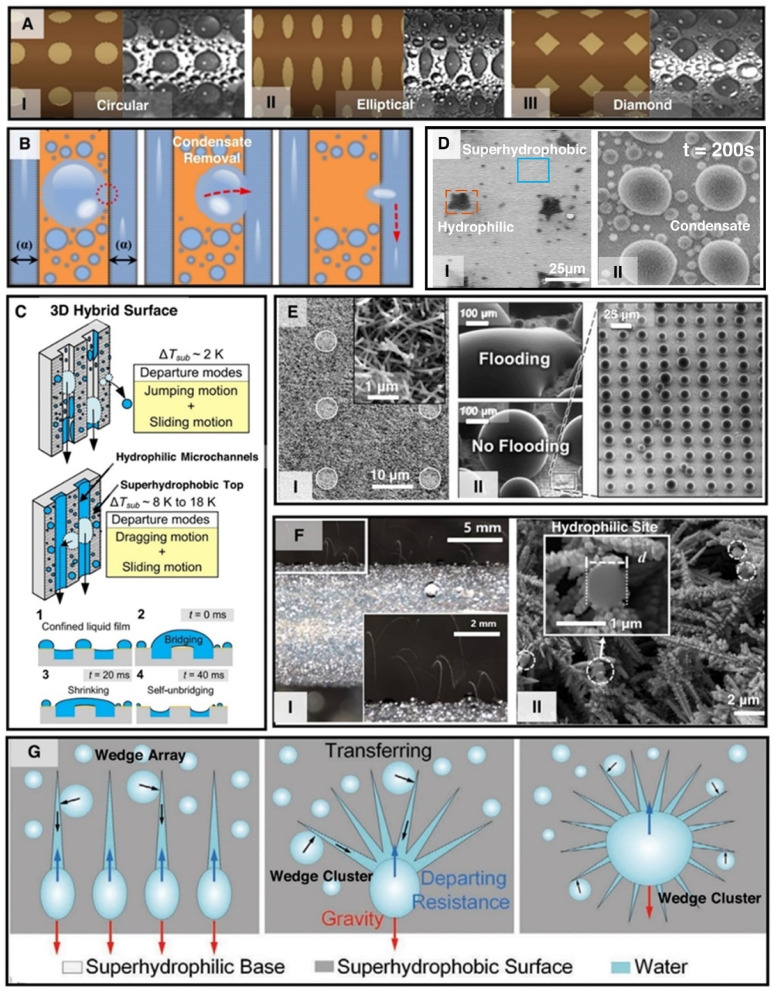
(A) Schematic of varying geometric shapes and corresponding image of steam condensation on (I) circle-shaped patterns, (II) ellipse-shaped patterns, and (II) diamond-shaped patterned hybrid wettability copper tubes.^259^ Reproduced with permission from ref. 259. Copyright 2022 Elsevier. (B) Concept of droplet migration mechanism between two different wettability regions.^205^ Reproduced with permission from ref. 205. Copyright 2017 Elsevier. (C) Schematic diagrams of condensate droplet departure modes and condensate self-unbridging mechanism on 3D hybrid surfaces consisting of hydrophilic microchannels within superhydrophobic surface.^214^ Reproduced with permission from ref. 214. Copyright 2016 Cell Press. (D) (D-I) Top-view SEM images of a stamped hybrid surfaces showing superhydrophobic background and stamped hydrophilic spots. (D-II) ESEM top view images of water vapor condensation on the stamped hybrid surface showing preferential nucleation of condensate on the hydrophilic spots.^256^ Reproduced with permission from ref. 256. Copyright 2020 American Chemical Society. (E-I) SEM image of surfaces with mixed wettability, comprising a superhydrophobic nanostructured surface patterned with a rectangular array of circular superhydrophilic microscale islands.^252^ (E-II) ESEM images of condensation on superhydrophobic and hybrid surfaces at high supersaturations (*S* = 1.54).^252^ Reproduced with permission from ref. 252. Copyright 2016 American Chemical Society. (F) A hybrid wettability tube consisting of biphilic nanomorphology exhibiting (I) jumping-droplet enhanced condensation mode enabled by (II) a grass-like nanostructured superhydrophobic surface patterned with nanoscale hydrophilic sites as shown in the SEM image.^255^ Reproduced with permission from ref. 255. Copyright 2020 Elsevier. (G) Schematic illustrating the self-driven condensate droplet transport enhanced dropwise condensation on hybrid superwetting surface with different superhydrophilic wedge and cluster combinations on a superhydrophobic base surface.^257^ Reproduced with permission from ref. 257. Copyright 2022 American Chemical Society.

To enhance condensation heat transfer and to prevent condensate flooding researchers have investigated many variations of hybrid surfaces with varying combination of surface wettability, *i.e.*, hydrophobic–hydrophilic,^253^ superhydrophilic–hydrophobic,^254^ superhydrophobic–hydrophilic^255,256^ and superhydrophilic–superhydrophobic.^257^ Furthermore, varying designs of hybrid patterned surfaces (*i.e.*, nature inspired patterns,^250^ branching topology,^258^ geometrical shapes,^259^ randomly distributed micro^250^ and nanopatterns^255^) and varying geometries of patterns (*i.e.*, grooved,^254^ wedge shaped,^257^ straight,^205^ square,^216^ circular,^260^ elliptical,^259^ diamond^259^) have also been investigated in recent years (Fig. 15).

Studies have shown that hydrophilic–hydrophobic finned surface can achieve superior performance with ∼40 kW m^−2^ K^−1^ condensation HTC in pure vapor conditions,^253^ while hydrophilic–superhydrophilic finned surface gives the best performance in the presence of non-condensable gases. By implementing straight and circular hybrid patterns on copper tubes researchers were able to achieve maximum condensation HTC of ∼85 kW m^−2^ K^−1^ which is 1.8 times the HTC of complete dropwise condensation at 9 K subcooling.^205^ However, implementing optimized circular patterns (Fig. 15A) yielded 50% higher HTC when compared to complete dropwise condensation at 9 K subcooling.^260^ In comparison, diamond shaped patterns (Fig. 15A) can provide 60% higher condensation HTC when compared to a complete hydrophobic surface and also have been shown to outperform circle and ellipse shaped patterns.^259^ These studies explored the effect of circular, parallel straight, and diamond shaped patterns of varying size and spacing where the patterns were more hydrophobic than the background and determined the optimized design to maximize the wettability contrast enabled condensate removal (Fig. 15B). A recent study has shown that a 3D hybrid surface (Fig. 15C), consisting of hydrophilic microchannels in superhydrophobic Si nanowire surface, can achieve maximum condensation HTC of ∼53 kW m^−2^ K^−1^ at ∼8 K subcooling.^214^ The 3D hybrid architecture prevents surface flooding at higher subcooling by confining the liquid-film thickness and enabling self-removal of liquid bridges formed on the surface (Fig. 15C). Studies have shown that implementation of constructal-like hybrid wettability patterns which presents a branching topology makes the condensate convergence and departing process more efficient, increasing condensate collection by 30%.^258^

Researchers have shown that hybrid wettability patterns at micro and nanoscale can enhance condensation heat transfer performance and prevent condensate induced flooding of superhydrophobic surfaces at higher supersaturation conditions (Fig. 15D–F).^252,255^ For example, microscale hydrophilic patterns can be utilized to impart spatial order of condensate through preferential nucleation and self-organization of coalescing droplets at high supersaturations (Fig. 15D and E). As a result, microscale hydrophilic patterned superhydrophobic surfaces have enhanced condensation heat transfer without condensate induced flooding when compared to flooded superhydrophobic surfaces at high supersaturations (Fig. 15E).^252^ Similarly, biphilic nanomorphology surfaces implemented on horizontal tube surfaces (Fig. 15F) have been shown to prevent condensate flooding by sustaining coalescence, droplet jumping and condensate self-removal.^255^ Thus biphilic nanomorphology enabled hybrid surface can achieve condensation enhancements resulting in 123% higher condensate collection at 60% relative humidity when compared with uncoated surfaces.

Hybrid surfaces consisting of wedge-shaped superhydrophilic patterns on superhydrophobic backgrounds (Fig. 15G) can transfer condensate from the dropwise region to the filmwise region facilitating droplet departure and thus enhancing condensation heat transfer by 30% when compared to a uniform superhydrophobic surface.^257^

Most of the literature on hybrid surfaces incorporates visualization studies to investigate mechanisms for sustaining dropwise condensation, condensate flooding prevention and enhanced condensation. The majority of these studies have been conducted in the presence of varying degrees of non-condensable gases which makes the acquired heat transfer performance of these surfaces incomparable. The scarcity of comparable condensation performance results of these hybrid surfaces makes it imperative to conduct further rigorous and standardized experimental studies in pure vapor environments to achieve a better understanding of the efficacy of hybrid wettability surfaces.

### 4.4 Nanostructuring of additively manufactured surfaces for enhanced condensation

**Fig. 16:**
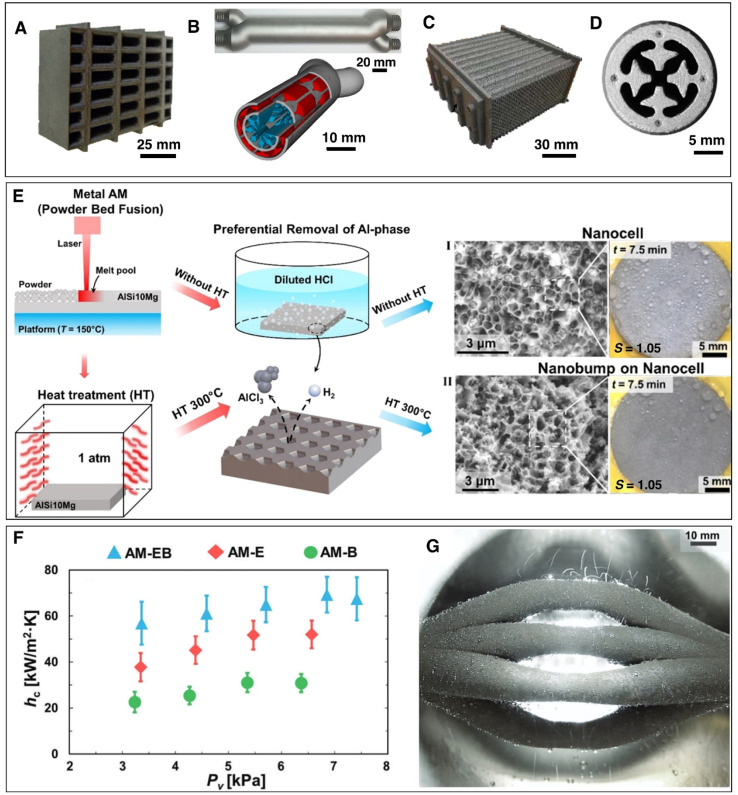
Metal additively manufactured (A) air-cooled heat exchangers,^264^ (reproduced with permission from ref. 264. Copyright 2018 Elsevier) (B) liquid cooled heat exchanger,^262^ (reproduced with permission from ref. 262. Copyright 2021 Cell Press) (C) porous ultra-compact heat exchangers,^261^ (reproduced with permission from ref. 261. Copyright 2020 Elsevier) and (D) tube with genetically optimized internal fins.^263^ (Reproduced with permission from ref. 263. Copyright 2021 Elsevier) (E) schematic illustrating the process for generating varying micro-/nano-structure morphologies on AM AlSi10Mg samples using metallurgical heat treatment and preferential removal of the Al-phase *via* chemical etching. Images showing the SEM of the micro/nanostructures on AM samples and their corresponding jumping droplet enabled condensation state at supersaturation of *S* = 1.05 for each surface treatment processes: (I) 7.5 min etched AM sample not heat treated (NHT) having dense nanocell structures; (II) 7.5 min etched AM sample heat treated at 300 °C (HT300) having dense nanocells with smaller scale dense nanobumps on the cell walls.^210^ Reproduced with permission from ref. 210. Copyright 2022 American Chemical Society. (F) Experimental steady-state condensation heat transfer coefficient as a function of saturated vapor pressure showing enhanced performance of two-tier nanostructured AM surface (AM-EB) compared to single-tier nanostructured AM surfaces (AM-E and AM-B).^201^ (G) Enhanced jumping-droplet condensation on the two-tier nanostructured AM heat exchanger at *P*_v_ = 6.5 kPa.^201^ Reproduced with permission from ref. 201. Copyright 2022 Wiley.

Additive manufacturing (AM) of metal heat exchangers has gained significant popularity in heat transfer and thermal management applications due to its design flexibility and ability to manufacture complex compact components, not achievable by conventional manufacturing techniques.^261–263^ Over the last decade researchers have shown implementation of additive manufacturing for the development of air-cooled heat exchangers,^264^ liquid cooled heat exchangers,^262^ porous ultra-compact heat exchangers,^261^ three-dimensional pin fins,^265^ and tubes with genetically optimized internal fins^263^ for enhanced heat transfer performance (Fig. 16A–D).

Recent studies have focused on synergistic combination of micro and nanostructuring of AM metal surfaces along with its design flexibility to further enhance condensation performance.^201,210,266^ Due to significantly different elemental composition of AM materials along with the AM laser melting and rapid solidification processes, as fabricated AM surfaces consist of unique sub-grain structures compared to conventionally manufactured metal surfaces.^267–269^ Researchers have demonstrated that these unique sub-grains have unveiled the possibility of generating unique micro and nanostructures by varying heat and chemical treatment of AM surfaces. For example, a recent study has demonstrated that for AlSi10Mg AM alloy, different combination of post processing methodology utilizing optimized heat treatment and chemical etching can yield unique micro/nanostructured surfaces (Fig. 16E).^210^ These nanostructures *i.e.*, nanocells, nanocell with nanobumps and microsteps can be utilized to develop superhydrophobic surfaces with varying degrees of droplet adhesion and condensate droplet repellency. The study compared the jumping droplet condensation performance of a conventional boehmite nanostructured surface with an AM non-heat treated etched (nanocells) and heat-treated etched (nanocell with nanobumps) AM surface. The structures with second-tier nanobumps generated by heat treatment at 300 °C followed by chemical etching showed the best performance exhibiting jumping droplet condensation with condensation HTC of ∼85 kW m^−2^ K^−1^ even at high supersaturation of *S* = 1.05. In comparison, the single tier nanocellular structured AM surface and the single tier boehmite nanostructured conventional aluminum surface failed to sustain jumping droplet condensation in similar conditions. The study showed that the second-tier cellular structured AM surfaces can sustain jumping droplet condensation by preventing lateral spreading of condensate droplets at higher nucleation and growth rates. This was further verified by another study where condensation performance of three large scale AM tubes consisting of single tier boehmite (AM-B), single tier nanocells (AM-E), two tier nanocells with boehmite (AM-EB) were compared (Fig. 16F).^201^ Unlike the single-tier nanostructured surfaces, two-tier nanostructured AM-EB surface sustained jumping droplet condensation even at high supersaturation of *S* = 1.8 at 7.4 kPa pure vapor pressure and showed the highest condensation HTC of ∼65 kW m^−2^ K^−1^ compared to the other surfaces and ∼6 times higher than filmwise condensation. This enhanced condensation was further shown on an AM two-tier nanostructured compact monolithic heat exchanger (Fig. 16G) proving the applicability of scalable nanostructuring method on unconventional AM heat exchangers.

### 4.5 Rational design of micro and nanostructures for enhanced flow condensation

Internal flow convective condensation can be described as condensation where a pure vapor or liquid–vapor mixture at some prescribed quality enters the test section and heat is removed from the walls allowing the vapor or mixture to condense. The majority of prior research that applies micro and nanostructuring to condenser surfaces has only focused on external quiescent condensation. Few studies have explored the implementation of these structures for internal flow convective condensation.

**Fig. 17:**
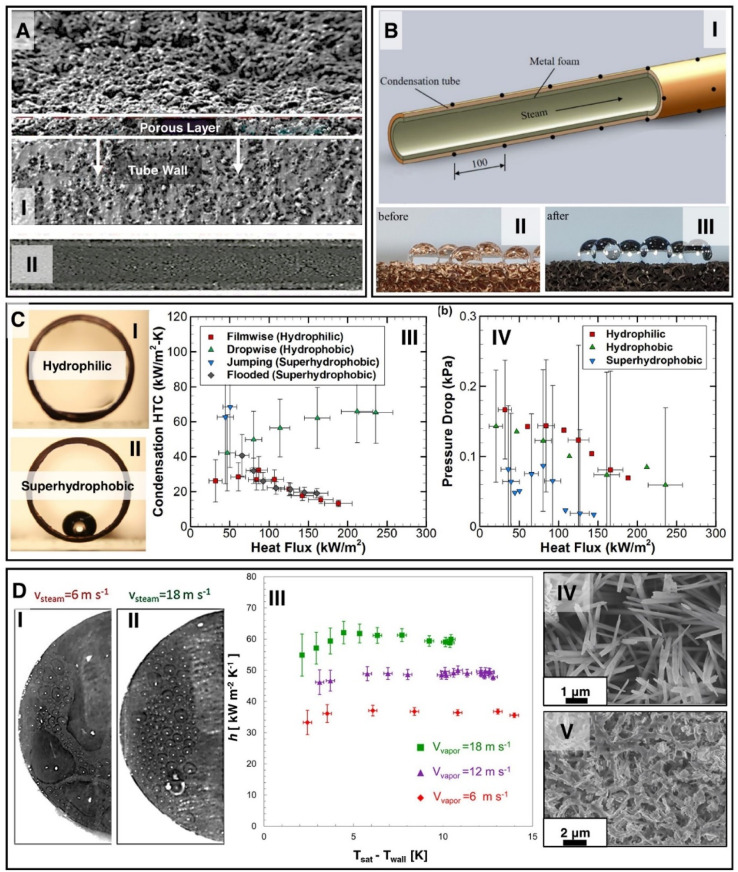
(A) SEM image of sintered porous coated tube: (I) axial view, (II) split view of the wire-electrode cut tube.^278^ Reproduced with permission from ref. 278. Copyright 2022 Elsevier (B) Schematic showing (I) a condensation test tube section filled with metal foam, (II) suspended water droplet showing the wetting state of the foam before treatment, and (III) after hydrophobic treatment.^274^ Reproduced with permission from ref. 274. Copyright 2019 Elsevier. Cross-section images of water droplets in (C-I) hydrophilic and (C-II) superhydrophobic condenser tube samples.^149^ (C-III) Estimated condensation heat transfer coefficient (HTC) for the different modes of condensation on the hydrophilic, hydrophobic and superhydrophobic surfaces.^149^ Reproduced with permission from ref. 149. Copyright 2020 Elsevier (C-IV) Pressure drop of the vapor and condensate flow through the test section, for the different condensation modes corresponding to the hydrophilic, hydrophobic and superhydrophobic surfaces.^149^ Reproduced with permission from ref. 149. Copyright 2020 Elsevier. Effect of vapor shear velocity on droplet size during DWC on a superhydrophobic copper sample at (D-I) *v*_steam_ = 6 m s^−1^ and (D-II) *v*_steam_ = 18 m s^−1^. Surface subcooling was 3.5 K.^275^ (D-III) Heat transfer coefficient dependence on the wall subcooling during DWC on a superhydrophobic copper sample at three different vapor velocities.^275^ SEM images of the superhydrophobic Cu(OH)_2_ nanowire sample (D-IV) before and (D-V) after five days of steam condensation experiments.^275^ Reproduced with permission from ref. 275. Copyright 2013 American Chemical Society.

Internal flow condensation enhancements are of great interest, as it has the potential to increase the efficiency in many applications *i.e.*, air-cooled coils for refrigeration,^270^ heat exchangers for thermosyphons used in electronics thermal management,^271^ and steam condensers in power plants.^272^ Internally functionalized copper tubes fabricated with infusion of a metal powder slurry and sintering afterwards (Fig. 17A) were shown to enable a 33% to 45% improvement in flow condensation HTC of R410a when compared to smooth tubes.^201^ For condensation heat transfer of R141b, implementation of copper oxide nanostructures was able to provide a 16.67% enhancement compared to the original copper channel with contact angle of 12.8°, which was attributed to the capillary force accelerating the condensate liquid when the mass flux was less than 400 kg (m^2^ s)^−1^.^273^

For steam condensation applications, a tube with a metal foam internal surface (Fig. 17B) was fabricated followed by surface oxidation and chemical modification with immersion in hydrochloric acid.^274^ The result is a metal foam with hydrophobic characteristics which can range from 10 PPI to 20 PPI. The study showed after hydrophobic coating metal foam tube could increase the flow condensation heat transfer performance and reduce the pressure drop compared to untreated metal foam tube.

A recent study has characterized the heat transfer and pressure drop performance of CuO nanostructured superhydrophobic condenser tube during internal flow condensation (Fig. 17C).^149^ The experimental results showed superhydrophobic surface had the highest HTC (≈68 kW m^−2^ K^−1^) at a lower heat flux (≈50 kW m^−2^). At a higher heat flux superhydrophobic surface transitioned to flooded condensation mode and exhibited HTC similar to that of filmwise condensation (≈26 kW m^−2^ K^−1^). However, dropwise condensation on hydrophobic surface reached maximum HTC (≈65 kW m^−2^ K^−1^) above ≈ 250 kW m^−2^ heat flux. The study also showed that for all the tubes the HTC increased with inlet vapor velocity and superhydrophobic surface exhibited lower pressure drop compared to hydrophobic and hydrophilic tubes. Another study investigated the condensation heat transfer performance of Cu(OH)_2_ nanowires under the stringent flow condensation conditions of saturated vapor at 110 °C saturation temperature up to a high heat flux ∼600 kW m^−2^ at 10 K subcooling and 18 m s^−1^ vapor velocity (Fig. 17D).^275^ The study showed that with an increase in the vapor velocity, the size of the departing droplets becomes smaller and enhances heat transfer performance on the superhydrophobic surface. Furthermore, the superhydrophobic surface showed enhanced condensation up to 5 days before nanostructure degradation resulting in transition of condensation mode to film condensation on the 6th day. Previous studies on external quiescent condensation have also reported similar degradation of structured surface where collapse and breakage of nanograss like structures deteriorated the heat transfer performance during long-term application.^199^ Further future work should be conducted in the future to develop robust and durable nanostructures for long term flow condensation application and to acquire a better understanding of the internal condensation mechanism. Investigations involving better visualizations can offer additional insights into temporal transition from jumping droplet condensation to filmwise condensation during internal forced convective vapor flow. Superhydrophobic surfaces can degrade during long term condensation due to degradation of structures or delamination of low energy coating.^275,276^ To address this concern, recent studies have focused on the development of robust micro and nano structures as well as development of durable low energy coatings with highly adhesive interfaces.^194,276^ Micro and nanostructures enabling jumping droplet condensation during internal flow condensation has been looked at in more details from a modeling perspective. Comprehensive modeling of jumping droplet condensation during internal flow condensation has been utilized to further elucidate the effects of droplet size, heat flux, droplet jumping location, vapor mass flux and pipe radius on droplet trajectory, heat transfer performance and pressure drop.^277^

## 5. Machine learning methods for boiling and condensation

Recently, there has been a steady rise in the use of machine learning (ML) techniques for prediction and characterization of complex two-phase heat transfer problems such as condensation and boiling.^279–283^ These techniques have shown a promising pathway for enhancing the performance of predictive models in these complex physical phenomena and have also helped with better feature extraction and understanding of the underlying physics. Many studies have focused on using ML models to substitute the empirical or semi-empirical correlations developed for pressure drop and heat transfer estimation of internal condensation and boiling. Supervised ML models such as Artificial Neural Network (ANN), Decision Tree, Random Forest, *K*-nearest neighbors regression (KNN-regression), Adaptive boosting (AdaBoost), and Support Vector Machine (SVM) have been developed as regression models to estimate the pressure drop and heat transfer coefficient in internal boiling and condensation processes.^284–289^ Qiu *et al.* built an ANN model using 16953 data points from 50 sources to predict internal flow boiling heat transfer in mini/micro-channels.^286^ The best ANN model had 7 hidden layers with number of units at each layer varying from 10 to 75 and it was trained only on dimensionless parameters including bond number (Bd), boiling number (Bo), convection number (Co), Froude number (Fr), Peclet number (Pe), Prandtl number (Pr), Reynolds number (Re), Suratman number (Su), and Weber number (We). This model achieved mean absolute error (MAE) of 14.3% over the training dataset which is almost half the MAE of empirical correlations. The MAE metric is defined by eqn (1), where *N* is the number of data points, *h*_exp_ and *h*_pred_ are the experimentally measured and predicted heat transfer coefficients.1
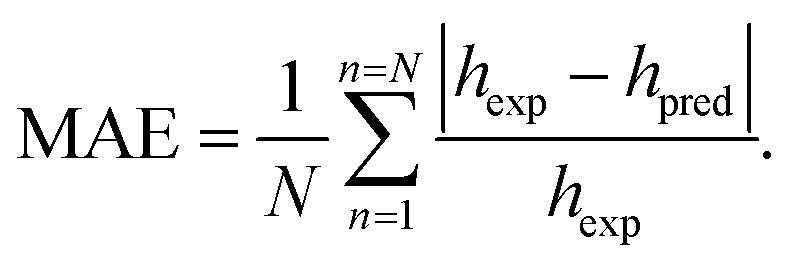


However, the model accuracy diminished slightly and drastically when testing on unseen data with working fluid included in the training dataset and unseen data with working fluid out of the training dataset, respectively. These results demonstrate that although the model was doing a good job interpolating from the data and attaining higher accuracy than empirical correlations, it failed in extrapolation on unseen working fluids with different properties. The same methodology was used to build 4 models of ANN, AdaBoost, Gradient Boosting, and Random Forest over 4882 data points from 37 sources to predict internal flow condensation heat transfer in mini/micro-channels with ANN showing the lowest MAE (6.8%) when testing on training dataset.^285^ This value is almost 20% lower than the MAE of the state-of-the-art correlation developed for flow condensation HTC. However, the model performed poorly on unseen data where the fluid information was not available in the training dataset and MAE increased to 77%, suggesting strong dependency to prior data. This is due to the lack of understanding of the underlying physics behind these models, making them only excellent regression models. Visual data could provide more insights into phase change problems which has led to development of more universal models. However, recording visual data from flow boiling and condensation systems is inherently challenging, and data is limited.

Visual data are more prevalent in pool boiling and external condensation processes. Recently, more studies have focused on developing deep learning based models using visual data for pool boiling heat flux estimation.^290–292^ One such example was data collection from pool boiling experiments using a commercialized DSLR camera and training of a convolutional neural network (CNN) to predict heat flux in nucleate boiling regime.^290^ The CNN model was able to capture bubble morphology which encoded extensive information about the heat flux. The drawback of this model was the single geometry configuration of the data used in training which lessened model universality. In another study, a high-speed camera was used to acquire 2000 frames per second (fps) images from pool boiling experiments to develop a physics-informed learning framework^8^ by combining an image recognition network (VGG16)^293^ with a segmentation network (Mask R-CNN).^294^ The image recognition network was used for hierarchical image feature extraction and the segmentation network was used to extract physical features such as bubble size and count. The outputs of these networks were passed through another multi-layer perceptron network to attain heat flux during the boiling as the final output. Their framework successfully predicted the steady-state and transient heat fluxes on boiling curve for heat fluxes up to 100 W cm^−2^.

In addition to the CNN based model developed for pool boiling heat flux estimation, recurrent neural networks could also be used to take into account the boiling process variation with time to better perceive the bubble dynamics and possibly predict the bubble dynamics in future frames which could be used for early detection of boiling crisis. A model developed using a bidirectional long short-term memory (BiLSTM) was able to predict bubble morphologies for pool boiling using the first few principal components of time-series data which were extracted using principal component analysis (PCA).^295^ Every single data consisted of 300 milliseconds (ms) of sequential data, from which the first 200 ms was used as the input and the next 100 ms as the output and the network was trained to predict the transient behavior of the principal components during the 100 ms period. The dominant frequency and the amplitude of the dominant frequency obtained from Fast Fourier Transform (FFT) of the predicted principal components were shown to be strong indicators of the onset of the boiling crisis. In addition to visual techniques, acoustic techniques have been shown to accurately predict CHF in pool boiling, through an increase in peak frequency on transitioning to CHF.^296^ Training of a CNN's with acoustic emissions have also been recently utilized for identification of boiling regimes such as natural convection, nucleate boiling, and transition boiling.^297^

Despite the presence of several studies of boiling, visual data along with deep learning models have been less explored for external condensation. A few recent works have however shown great potential in application of these techniques for characterization of external condensation. A vision-based deep learning framework consisting of object detection, tracking, and data processing modules for external condensation images has been recently proposed.^298^ Using this framework on condensation visual data, physical descriptors such as droplet growth rate, droplet distribution, and heat flux were extracted. Statistical analysis of the segmented droplet images during condensation on hydrophobic and superhydrophobic flat surfaces enabled studying transient measurement of heat flux and the effects of single droplet heat transfer rate and the droplet size distribution on the total heat transfer rate from the surface. In another study, a methodology was proposed to measure condensation heat flux (with uncertainty less than 10%) using condensation videos by detecting and counting falling droplets from condensing tubes,^299^ using an object detection network called EfficientDet.^300^ By eliminating the need for temperature sensors, the proposed method achieved lower uncertainty compared to the conventional experimental methods. Furthermore, successful application of this methodology enabled local heat transfer measurements on tubes having axially varying surface properties resulting in different heat transfer rates at each location.

A summary of recent studies focusing on learning-based models for condensation and boiling is shown in Table 1. Most studies have focused on development of supervised models to replace the empirical correlation and have shown strong capability for regression on training datasets. However, these lack generalizability for extrapolative purposes and suffer from low explainability.^280^ Intelligent-vision based methods could be used to extract meaningful and physical features from complex pool boiling and external condensation studies. In addition to extracting features, novel, low-cost, and robust characterization methods could also be developed using state-of-the-art deep learning algorithms along with visual data to replace complex and expensive experimental methods. These advanced methodologies have been mainly limited to pool boiling and external condensation due to higher visual data availability for these processes. With recent advances in data-driven models and learning-based computer vision algorithms and the recent deployments of these methods for thermofluidic sciences, application of these techniques to structured surface phase-change processes can greatly improve predictive ability and aid in future experimental design.

**Table 1:** Summary of recent applications of ML algorithms for phase-change heat transfer

Reference	ML model	Application	Dataset	Details
Qiu *et al.*^284^	ANN, Gradient Boosting, KNN	Flow boiling pressure drop	2878 data points from 21 sources. Inputs: 23 dimensionless numbers (Bd, Bo, Fr, Pr, Pe, Re, Su, We)	ANN had the best performance on training dataset. MAE 13.7% lower than empirical correlation. Poor performance when tested fluid not in training dataset
Zhou *et al.*^285^	ANN, AdaBoost, Random Forest, Gradient Boosting	Flow condensation heat transfer coefficient	4882 data points from 37 sources. Inputs: 22 dimensionless numbers (Bd, Co, Fr, Ga, Ka, Pr, Re, Su, We)	ANN had the best performance on training dataset. MAE 19.8% lower than empirical correlation. Performed poor when tested fluid not in training dataset
Qiu *et al.*^286^	ANN	Flow boiling heat transfer coefficient	16 953 data points from 50 sources. Inputs: 21 dimensionless numbers (Bd, Bo, Co, Fr, Pe, Pr, Re, Su, We)	MAE 13.1% lower than empirical correlation. Poor performed when tested fluid not in training dataset
Hughes *et al.*^287^	ANN, Support Vector Regression, Random Forest	Flow condensation pressure drop	4000 data points from 18 sources. Inputs: 8 dimensionless numbers (*x*, Re, Fr, Bo, We, AR)	Random Forest was the most accurate model with absolute average deviation of 3.4%
Zhu *et al.*^288^	ANN	Flow boiling heat transfer coefficient	1500 data points from authors experiments. Inputs: 20 dimensionless numbers (Co, Bd, Bo, Pr, Fr, Re, Su, We, Fa, *P*_R_, *X*)	MAE was 11.41%
Liang *et al.*^289^	ANN	Flow boiling heat transfer coefficient and wall temperature	531 data points from authors experiments. Inputs: 8 attributes (mass flow rate, thermal power, inlet pressure and temperature, direction, acceleration, tube inner surface area, helical coil diameter)	MAE was 7.4%
Hobold and Silva^290^	CNN	Nucleate boiling heat flux	84 096 image data inputs: grayscale frames of pool boiling at resolution of 320 by 240 pixels captured at 31 frames per second	MAE < 10. Real-time prediction possible and each prediction taking 142 ms on Raspberry 3 model B
Suh *et al.*^291^	Instance segmentation (Mass R-CNN) + object recognition CNN (VGG16) + multi-layer perceptron (MLP)	Pool boiling heat flux	3250 pool boiling image data captured at 2000 fps with resolution of 1024 × 1024 pixels	MAE was 6% for heat fluxes varying from 10 W cm^−2^ to 100 W cm^−2^. Real-time prediction was possible
Rassoulinejad-Mousavi *et al.*^292^	CNN	CHF detection in pool boiling	43 795 images of pool boiling experiments from 3 different sources	Model accuracy varied from 99% to 94% based on training sample size
Rokoni *et al.*^295^	PCA + RNN (LSTM)	CHF detection and bubble morphology in pool boiling	25 000 images of pool boiling experiments	Reduced-order future images during pool boiling estimated. Average structural similarity index measure (SSIM) between predicted image and the real image was 0.983
Suh *et al.*^298^	Mask R-CNN + object tracking (*k*-dimensional tree algorithm)	External condensation heat flux of flat surfaces	2460 images of dropwise condensation on flat surfaces	The mean average pixel error (MAPE) was 3%
Khodakarami *et al.*^299^	Object detection (EfficientDet) + object tracking	External condensation heat flux of tubes	350 images of falling droplets during external condensation on tubes captured at 30 fps with resolution of 1080 × 1920 pixels	The heat flux uncertainty <10%; smaller than past experimental methods for all conditions. No temperature measurement conducted

## 6. Conclusions and outlook

This paper presents a comprehensive review of the recent progress in the development of micro/nanostructured surfaces for enhanced boiling and condensation. Review of recent studies shows the development of numerous surface nanostructures for minimizing condensate droplet departure size by achieving enhanced jumping droplet condensation or enhanced droplet shedding on lubricant infused surfaces. Despite development of a plethora (>1000) micro and nanostructures, only a few recent studies have explored surface nanostructures that can exhibit sustainable jumping droplet condensation at higher supersaturation and subcooling conditions. Recent studies have shown implementation of nanostructure morphology which facilitates condensate droplet nucleation within interstructure spacing preventing excessive lateral spreading of the condensate enabling jumping droplet condensation even at higher supersaturation and subcooling. However, when it comes to enhanced condensation of low surface tension liquids, recent studies have shown that the nanostructure itself cannot sustain dropwise condensation due to interstructure flooding by nanoscale condensate droplets and requires infusion with low energy lubricants to enable the shedding of the condensate droplets from the low hysteresis lubricant surface. Many studies have focused on condensation durability of these micro and nanostructured surfaces and proposed rational designs for long-term condensation considering three aspects which includes mechanical robustness of the structures, durability of the low energy coating, and oil retention capability of lubricant infused surfaces.

When it comes to boiling enhancement, this review elucidated the role of structures in promoting bubble nucleation, preventing dry out and prolonging wicking anti-degeneration. Apart from the significant enhancements in HTC and CHF achieved by structured surfaces, the recent development of state-of-art *in situ* optical visualization techniques also enables the identification of previously unidentified three-phase contact line dynamics, advancing the fundamental understanding of the complex thermal transport process and bubble–structure interaction mechanisms. While many existing works focus on the understanding of bubble generation mechanisms on structures and their thermal performance, equally important are their robustness, durability and anti-degeneration characteristics required to sustain long-term (>5 years) operation. A recent study has demonstrated good anti-degeneration of structures through year-long wickability and boiling heat transfer measurements in water, however more studies are needed for other structures and working fluids.

Highly scalable, shape conformal and cost-effective structuring strategies are important criteria for industrial implementation. While structuring methods such as thermal oxidation, wet etching and electrochemical deposition have demonstrated these attributes, they are implemented only on copper and aluminum alloys. Development of similar structuring methods for other metals such as stainless steel and titanium are important to improve the efficiencies of condensers, boilers and evaporators facilities requiring good corrosion resistance or high temperature operations.

While most studies focus on the development of new and previously-unpublished structure morphologies, structuring strategies that can be implemented on internal surfaces and that are able to promote boiling or condensation, are compatible and durable with low surface tension fluids such as refrigerant and dielectric fluids, remain challenging and require further study. If successful, the development of these structures will have significant implications to a number of industries.

Studies have shown that optimization of structure designs across multitude of length scales opens the door to further enhancement in boiling and condensation performance. Recent studies have demonstrated this design methodology through the generation of structures consisting of nanopores, micro-dendrites and micropores (hundreds of nanometers to tens of micrometers) to form three-tier hierarchical surfaces achieving approximately 3X CHF enhancements in pool boiling. In future, it would be interesting to further explore the design space by combining macrometric features of millimeter length-scale with the micro- and nanostructures. Such optimization of strategies can be useful in regulating the vapor escape pathway with the appropriate placement of macro-fins while utilizing the micro/nanostructures as preferential bubble nucleation sites. Similarly, hierarchically nanostructured surfaces have been shown to provide higher condensation heat transfer enhancement and exhibit sustainable jumping droplet condensation even at extreme conditions. More work directed at comparison of microscale and nanoscale structures for specific applications such as condensation and boiling heat transfer enhancement needs to be performed to better summarize the effects of the two length scales.

Metal AM techniques have received significant attention as they possess great versatility to manufacture complex functional parts with complex macro-geometries and intricate millimeter or sub-millimeter features. Due to the unique layer-by-layer fabrication process, recent studies have demonstrated the potential of generating new and tunable micro and nano-architectures on AM alloys previously not found on conventionally produced metals. Although past investigations have demonstrated the exceptional performance of these structures in droplet repellency, condensation and anti-icing, their wicking and boiling characteristics remain unknown, and would be interesting to study in future.

Finally, much work has been devoted over the past few years to the development of data-driven models and learning-based computer vision algorithms in the field of thermofluidic sciences. Although a plethora of ML studies have been conducted for predicting heat transfer performance of conventional surfaces, our review shows there is a lack of machine learning based studies for evaluating micro and nanostructured surface performance. Application of these techniques to structured surface enabled phase-change processes can greatly improve predictive ability and aid in future experimental designs given the many choices of surface structures available.

## Conflicts of interest

There are no conflicts to declare.

## Supplementary Material
